# The structure and existence of solutions of the problem of consumption with satiation in continuous time

**DOI:** 10.1371/journal.pone.0216383

**Published:** 2019-05-09

**Authors:** Peter Smoczynski, Stan Miles

**Affiliations:** 1 Department of Mathematics and Statistics, Faculty of Science, Thompson Rivers University, Kamloops, BC, Canada; 2 Department of Economics, School of Business and Economics, Thompson Rivers University, Kamloops, BC, Canada; Universidad Catolica San Antonio de Murcia, SPAIN

## Abstract

With the help of the method of Lagrange multipliers and KKT theory, we investigate the structure and existence of optimal solutions of the continuous-time model of consumption with satiation. We show that the differential equations have no solutions in the *C*^1^ class but that solutions exist in a wider space of functions, namely, the space of functions of bounded variation with non-negative Borel measures as controls. We prove our theorems with no additional assumptions about the structure of the control Borel measures. We prove the conjecture made in the earlier literature, that there are only three types of solutions: I-shaped solutions, with a gulp of consumption at the end of the interval and no consumption at the beginning or in the interior; U-shaped solutions, with consumption in the entire interior of the interval and gulps at the beginning and the end; and intermediate (J-shaped) solutions, with an initial interval of abstinence followed by a terminal interval of distributed consumption at rates and a gulp at the end. We also establish the criteria that permit determination of the solution type using the problem’s parameters. When the solution structure is known, we reduce the problem of the existence of a solution to algebraic equations and discuss the solvability of these equations. We construct explicit solutions for logarithmic utility and CRRA utility.

## 1 Introduction

Recently, Baucells and Sarin [1, 2] described a new and interesting discrete-time model of consumer behavior: the satiation model. The psychological justifications of the model are explained in plain language in [3]. In [4, 5] this model was extended to continuous time, and the solutions were constructed for CRRA utilities.

The satiation model presented in [4, 5] is mathematically described as an optimal control problem (Problem 1) in this paper. In this problem the control (consumption *c*(*t*)) enters the evolution equation (Eq (2)) in this paper linearly and may be unbounded, hence the classical Pontryagin Maximum Principle (see [6]) is not directly applicable. For problems of this type, often called systems with impulsive controls, more general maximum principles are discussed in [7] and [8], and extensively in [9] and in papers quoted therein. All of these works use a solution-dependent change of the time variable which was introduced in [10]. In [11] the Bellman principle is extended to impulsive systems. In all of these works it is explicitly assumed that the measure controls have atoms. All of these general theories are very complex, hence we convert Problem 1 into a variational problem, and we use the simpler and more straightforward method of Lagrange multipliers. The approach presented in this paper uses very little beyond the finite-dimensional case described in [12]. Our method is simpler than any proof of a maximum principle, especially when unbounded controls are involved. The additional novelties of our approach are as follows: Instead of assuming a priori that the control measures have atoms, we prove that they do. In addition, we use no transformation of the time variable, and we do not require any additional differential equations (such as Eq (4.6) and later equations in [8]) to be satisfied by the atoms of the control measures.

In this work, Problem 1 is transformed into a problem that is easier to handle: Problem 3, with a different utility (see Eq (9)). We first prove that this problem has no solutions with continuously differentiable satiation *s*. Then we extend the solution space to the BV space (the space of functions of bounded variation). This implies that we must allow the consumption *c*, the controlling variable, to be a general Borel measure; it could even be as exotic as the derivative of the Cantor function. An important lemma (Lemma 23) is proved using only the assumption that *c* is a non-negative Borel measure.

We conduct a detailed investigation of the structure of optimal solutions for general utilities. Our general results are proved under the following assumptions: (i) the original utility *V* and the transformed utility are both concave down, or −*V* and −*V*_*S*_ are both convex in the classic sense; and (ii) additional barrier conditions (Eqs (36) and (37)) hold. Further, we assume two pairs of inequalities, where one pair (Eqs (32) and (40)) corresponds to a high future discount *n*, and the other pair (Eqs (33) and (41)) corresponds to a low future discount *n*. Each pair is sufficient for the existence of solutions; however, the corresponding solutions differ remarkably. In the case of a high future discount, all the consumption must take place in a single gulp, or burst, at time *t* = 0, while in the case of a low future discount a terminal gulp of consumption at *t* = *T* is necessary. In [4] the proofs of many theorems use an additional assumption: that there are no gulps of consumption in (0, *T*). We prove (in Theorems 21 and 25) that this assumption is correct, namely, that the measure *c* is continuous relative to Lebesgue measure in (0, *T*), which means there are no gulps of consumption there, and that gulps of consumption can occur only at the endpoints of the interval [0, *T*]. We prove that at most one interval with non-zero consumption is possible and that at most one interval with no consumption is possible. This is done in Theorem 21 for the case of a large future discount and in Theorem 25 for a small future discount. We extend an earlier observation in [4], which was stated only for special cases of utilities and selected parameters, to general utilities and prove that under one of the aforementioned pairs of inequalities, which essentially corresponds to a large future discount, the only type of solution is that in which all the consumption takes place in a single gulp at *t* = 0 (as discussed in Section 6.1), while under the other pair of inequalities, which essentially corresponds to a small future discount, there are three types of solutions: J-shaped ones that correspond to poor or over-satiated agents; U-shaped ones that correspond to rich or under-satiated agents, with two gulps of consumption, one at the beginning and the other at the end; and intermediate solutions with an initial period of abstinence, a terminal gulp of consumption, and a terminal interval of non-gulpy consumption.

The general theory presented in Section 5 is used in Section 8 to reduce the problem of the existence of solutions to simple non-differential equations, and the solvability of these equations is proved. In the case of CRRA utility (Section 8.2), only one of these equations requires numerical methods, while in the case of logarithmic utility all of them are solved explicitly (Section 8.1).

## 2 The problem and preliminaries

In this paper, we address the following problem, which is a continuous-time version of the satiation problem in [1]:

**Problem 1** *Let V be a concave-down, twice differentiable function of s. Maximize the functional (the sum of instantaneous utilities* $\frac{dV}{ds}\cdot c\cdot dt$)

$$\begin{array}{c} U_{S}=\int_{\left[0,T\right]}e^{-n\cdot t}\cdot \frac{dV}{ds}(s(t))\cdot c(t)\cdot dt \end{array}$$ (1)

*under the following constraints*:

$$\begin{array}{ccc} \frac{ds}{dt} & = & \varphi \cdot c-\gamma \cdot s \end{array}$$ (2)

$$\begin{array}{ccc} s(0) & = & s_{0} \end{array}$$ (3)

$$\begin{array}{ccc} \{{subscript}_{0}\;addedd\;here\}s_{0} & \geq & 0 \end{array}$$ (4)

$$\begin{array}{ccc} c & \geq & 0 \end{array}$$ (5)

$$\begin{array}{ccc} \int_{\left[0,T\right]}e^{-r\cdot t}\cdot c(t)\cdot dt & = & W \end{array}$$ (6)

In this model, *s*(*t*) is the satiation level caused by consumption *c*(*t*), and *W* is the initial wealth; *n*, *r*, *γ*, and *φ* are positive parameters representing respectively the agent’s discount rate, the risk-less interest rate, the satiation decay rate, and the satiation generation rate caused by consumption *c*.

**Remark 2** *In Problem 1 we deliberately do not specify the space in which we search for solutions*. *It is more convenient to do this in the transformed Problem 3*. *However, in Section 3 we argue that the classical space used in Problem 3 does not contain any solutions of the problem*. *The proper space of BV functions is described in Section 4 and is used in the final version of the problem (Problem 10)*.

It is helpful to eliminate *c*(*t*) from (1). By constraint (2),

$$\begin{array}{c} c=\frac{1}{f}\cdot (\frac{ds}{dt}+\gamma \cdot s) \end{array}$$

Substituting this into (1) and then using integration by parts, we obtain an equation for the functional *f* ⋅ *U*_*S*_ in which the consumption function *c* does not explicitly appear:

$$\begin{array}{ccc} f\cdot U_{S}(s(t)) & = & \int_{\left[0,T\right]}e^{-n\cdot t}\cdot \frac{dV}{ds}(s(t))\cdot (\frac{ds}{dt}+\gamma \cdot s)\cdot dt \end{array}$$ (7)

$$\begin{array}{ccc} & = & e^{-n\cdot T}\cdot V(s(T))-V(s_{0})+\int_{\left[0,T\right]}e^{-n\cdot t}\cdot V_{S}(s(t))\cdot dt, \end{array}$$ (8)

where

$$\begin{array}{c} V_{S}(s)=n\cdot V(s)+\gamma \cdot s\cdot \frac{dV}{ds}(s). \end{array}$$ (9)

The following will be used later, in the proofs of Propositions 8 and 14 and Theorem 21:

$$\begin{array}{c} \frac{dV_{S}}{ds}(s)=(n+\gamma \cdot (1-\alpha ))\cdot \frac{dV}{ds}, \end{array}$$ (10)

where

$$\begin{array}{c} \alpha =\alpha (s)=-\frac{s\cdot \frac{d^{2}V}{ds^{2}}}{\frac{dV}{ds}} \end{array}$$ (11)

The expression for *α*(*s*) looks very similar to the expression for relative risk aversion. The term *relative risk aversion* is appropriate in the context of investments, where every investment carries some risk. However, in this paper the risk of consumption is ignored, hence the term *relative satiation aversion* seems more appropriate. Thus we will call *α*(*s*) the relative satiation aversion. The relative satiation aversion for *V*_*S*_ is denoted by *α*_*S*_:

$$\begin{array}{c} \alpha_{S}(s)=-\frac{s\cdot \frac{d}{ds}((n+\gamma \cdot (1-\alpha ))\cdot \frac{dV}{ds})}{(n+\gamma \cdot (1-\alpha ))\cdot \frac{dV}{ds}}=\alpha -\frac{\gamma }{n+\gamma \cdot (1-\alpha )}\cdot s\cdot \frac{d\alpha }{ds} \end{array}$$ (12)

Similarly to the method used in obtaining (7), we can transform the wealth constraint (6) with the help of integration by parts:

$$\begin{array}{c} f\cdot W=e^{-r\cdot T}\cdot s(T)-s_{0}+(\gamma +r)\cdot \int_{\left[0,T\right]}e^{-r\cdot t}\cdot s(t)\cdot dt \end{array}$$ (13)

We assume that *V*_*S*_ given by (9) is an increasing, concave-down function of the satiation *s*. In Section 8.2 it is demonstrated that this is always the case for CRRA utility. Therefore, Problem 1 can be replaced by the following problem:

**Problem 3 (The smooth version)** *Maximize the functional f* ⋅ *U*_*S*_ *in* (8) *on the space of all continuously differentiable functions s* ∈ *C*^1^ ([0, *T*] → *R*) *that satisfy the constraints* (4), (5), and (3), *and constraint* (13) *with W* > 0, *under the assumption that V and V*_*S*_ *are increasing, concave-down, twice continuously differentiable functions of s*.

**Remark 4** *If V is an increasing function, then it follows from* (10) *that V*_*S*_ *is increasing if*

$$\begin{array}{c} n+\gamma \cdot (1-\alpha )>0 \end{array}$$ (14)

*Downward concavity of V*_*S*_ *requires that α*_*S*_ > 0, *and downward concavity of V requires that α* > 0. *Therefore, these two conditions are assumed to be satisfied in everything that follows*.

One of the objectives of this paper is to discuss the solvability conditions for Problem 3. Necessary and sufficient conditions are obtained by generalization of the Lagrange multipliers (Karush–Kuhn–Tucker (KKT) theory for a finite number of dimensions; see [12]). We first note an important consequence of (5):

**Proposition 5** *If* (4) *holds and* (5) *holds for all t* ∈ [0, *T*], *then s*(*t*) > 0 *for all t* ∈ [0, *T*].

**Proof**. The solution of Eq (2) is

$$\begin{array}{c} s(t)=e^{-\gamma \cdot t}\cdot (s_{0}+f\cdot \int_{\left[0,T\right]}e^{\gamma \cdot t_{1}}\cdot c(t_{1})\cdot dt_{1}), \end{array}$$ (15)

hence *s*(*t*) > 0 for *t* ≥ 0, thanks to (4) and (5).

**Remark 6** *Later it is shown that s*(*t*) > 0 *in* (0, *T*] *when W* > 0 *even if s*_0_ = 0; *see Remark 36*.

Following the finite-dimensional KKT theory (see [12, 13]), we introduce the Lagrangian functional

$$\begin{array}{ccc} f\cdot \Lambda (s(t)) & = & f\cdot U_{S}(s(t))+\lambda_{W}\cdot (W-\int_{\left[0,T\right]}e^{-r\cdot t}\cdot (\frac{ds}{dt}+\gamma \cdot s)\cdot dt)\\ & & \;+\int_{\left[0,T\right]}\lambda_{c}\cdot [\frac{ds}{dt}+\gamma \cdot s]\cdot dt \end{array}$$ (16)

Here λ_*W*_ ∈ *R* is the Lagrange multiplier that corresponds to constraint (13), and λ_*c*_ ∈ *C*^1^ ([0, *T*] → *R*) corresponds to (5). The KKT method also involves additional constraints on λ_*c*_(*t*):

$$\begin{array}{c} 0=\lambda_{c}\cdot c=\lambda_{c}\cdot \frac{1}{f}\cdot (\frac{ds}{dt}+\gamma \cdot s),\;\lambda_{c}\geq 0. \end{array}$$ (17)

Since *s*(*t*) and λ_*c*_(*t*) are assumed to be differentiable on [0, *T*], they are continuous on that interval. Thus after integration by parts, the functional (16) takes the following form:

$$\begin{array}{ccc} f\cdot \Lambda (s(t)) & = & e^{-n\cdot T}\cdot V(s(T))-V(s_{0})-\lambda_{W}\cdot (e^{-r\cdot T}\cdot s(T)-s_{0})\\ & & \;+(\lambda_{c}(T)\cdot s(T)-\lambda_{c}(0)\cdot s_{0})\\ & & \;+\int_{\left[0,T\right]}[e^{-n\cdot t}\cdot V_{S}(s(t))-\lambda_{W}\cdot e^{-r\cdot t}\cdot (\gamma +r)\cdot s\\ & & \;\;\;+(-\frac{d\lambda_{c}}{dt}+\lambda_{c}\cdot \gamma )\cdot s]\cdot dt+\lambda_{W}\cdot W \end{array}$$ (18)

The first-order conditions for an extremum are obtained as follows: If *s*(*t*) is an optimal solution and Δ*s*(*t*) ∈ *C*^1^([0, *T*] → *R*) with Δ*s*(0) = 0 (hence for every real number *ε*, *s* + *ε* ⋅ Δ*s* satisfies the initial condition (3): *s*(0) + *ε* ⋅ Δ*s*(0) = *s*_0_), then

$$\begin{array}{ccc} \int_{\left[0,T\right]}\frac{\partial \Lambda }{\partial s()}\cdot \Delta s(t)\cdot dt & = & \left[e^{-n\cdot T}\cdot \frac{dV}{ds}(s(T))-\lambda_{W}\cdot e^{-r\cdot T}+\lambda_{c}(T)]\cdot \Delta s(T\right)\\ & & \;+\int_{\left[0,T\right]}[e^{-n\cdot t}\cdot \frac{dV_{S}}{ds}(s(t))-\lambda_{W}\cdot e^{-r\cdot t}\cdot (\gamma +r)\\ & & \;\;+(-\frac{d\lambda_{c}}{dt}+\lambda_{c}\cdot \gamma )]\cdot \Delta s(t)\cdot dt, \end{array}$$

which must be 0, hence also $\frac{\partial \Lambda }{\partial s()}=0$ by the DuBois–Reymond lemma, as Δ*s*(*t*) is arbitrary (except for Δ*s*(0) = 0, which follows from (3)). This leads to the following set of first-order conditions:

At the boundary (i.e., at *t* = *T*),

$$\begin{array}{c} e^{-n\cdot T}\cdot \frac{dV}{ds}(s(T))-\lambda_{W}\cdot e^{-r\cdot T}+\lambda_{c}(T)=f\cdot \frac{\partial \Lambda }{\partial s(T)}=0. \end{array}$$ (19)

In the interior of the interval (i.e., for *t* ∈ (0, *T*)),

$$\begin{array}{c} e^{-n\cdot t}\cdot \frac{dV_{S}}{ds}-\lambda_{W}\cdot e^{-r\cdot t}\cdot (\gamma +r)+(-\frac{d\lambda_{c}}{dt}+\lambda_{c}\cdot \gamma )=f\cdot \frac{\partial \Lambda }{\partial s()}=0. \end{array}$$ (20)

KKT theory guarantees that if Problem 3 has a solution *s*, then that solution is the first of the three entities in [*s*(*t*), λ_*c*_(*t*), λ_*W*_] that make up the solution of the following problem:

**Problem 7 (The *C*^1^ version)** *Find* [*s* (*t*), λ_*c*_ (*t*), λ_*W*_] ∈ *C*^1^ ([0, *T*] → *R*) × *C*^1^ ([0, *T*] → *R*) × *R that satisfies* (2), (20), *initial condition* (3), *terminal condition* (19), *constraints* (4) *and* (5), *constraint* (13) *with W* > 0, *and KKT conditions* (17).

We show in Section 3 that Problem 7 has no (everywhere differentiable) solution. The remedy, discontinuous functions with bounded variation, is described in Section 4. Problem 7 is reformulated as Problem 10 in the BV space. The sufficient conditions are derived in Theorem 11, and in Theorem 13 it is shown that the sufficent conditions are also necessary. The structure of these discontinuous solutions is investigated in greater detail in Section 6. Explicit solutions for logarithmic and CRRA utilities are constructed in Sections 8.1 and 8.2, respectively.

## 3 Nonexistence of continuously differentiable solutions

By assumption, *V* and *V*_*S*_ are concave down and all the constraints are linear, hence there can be no more than one solution. We will now show that the existence of a solution of the most tractable form (continuous on [0, *T*] and continuously differentiable on (0, *T*)) will lead to the contradictions described in the proof of the proposition below.

**Proposition 8** *If* (*r* − *n* + *γ* ⋅ *α*(*s*)) ≠ 0 *and* $\frac{dV}{ds}(s)\neq 0$ *for all s* ≥ 0, *then there is no continuously differentiable solution* [*s*, λ_*c*_, λ_*W*_] *of the boundary value problem (Problem 7)*.

**Proof**. Assume that there is a continuously differentiable solution. First, we assume that consumption *c*(*T*) > 0. Since *s* ∈ *C*^1^ ([0, *T*] → *R*), (2) implies that *c* is also continuous and therefore *c*(*t*) > 0 for all *t* in an open neighborhood of *T*. In this case, as a consequence of the first KKT condition in (17), we have λ_*c*_(*t*) = 0 for all *t* close to *T*, hence the terminal condition (19) and condition (20) with *t* = *T* take the following form:

$$\begin{array}{ccc} 0 & = & e^{-n\cdot T}\cdot \frac{dV}{ds}(s(T))-\lambda_{W}\cdot e^{-r\cdot T} \end{array}$$ (21)

$$\begin{array}{ccc} 0 & = & e^{-n\cdot T}\cdot \frac{dV_{S}}{ds}(s(T))-\lambda_{W}\cdot e^{-r\cdot T}\cdot (\gamma +r). \end{array}$$ (22)

One can eliminate λ_*W*_ by subtraction, obtaining, with the help of (10),

$$\begin{array}{c} 0=e^{-n\cdot T}\cdot [\frac{dV_{S}}{ds}(s(T))-\frac{dV}{ds}(s(T))\cdot (\gamma +r)]=(n-r-\alpha \cdot \gamma )\cdot \frac{dV}{ds}(s(T)). \end{array}$$

However, $\left(n-r-\alpha \cdot \gamma )\cdot \frac{dV}{ds}(s(T)\right)$ cannot be 0, by the premises of this proposition. Therefore, the assumption that *s*(*t*) is continuously differentiable, and that *c*(*T*) > 0, is false. The problem seems to be caused by the fact that Eq (20) has to be satisfied with *t* → *T*. We could try to get around this problem by assuming that there is no consumption at *t* = *T*, hence that λ_*c*_(*T*) ≠ 0, in which case we have one additional variable, λ_*c*_(*T*), that can help us to satisfy Eq (20) at *t* = *T*. If *c*(*T*) = 0 and *c*(*t*) > 0 for all *t* close to *T*, then λ_*c*_(*t*) = 0 for all these *t*, hence λ_*c*_(*T*) = 0 since λ_*c*_ is continuous in [0, *T*], so we do not have the additional variable λ_*c*_(*T*). In order to obtain it, we need to assume that there is some *T*_*e*_ < *T* such that *c*(*t*) = 0 for all *t* > *T*_*e*_ and *c*(*t*) > 0 for *t* ≤ *T*_*e*_. But in such a case the solution must be optimal in the interval [0, *T*_*e*_] and at *t* = *T*_*e*_ the same boundary condition must be satisfied, hence we obtain the same contradiction. Therefore, the assumption that *s*(*t*) is continuously differentiable is false.

Proposition 8 implies that Problem 1 does not have a continuously differentiable solution. Note, however, that all of the results proved in this section for continuous, almost everywhere differentiable functions (except for the nonexistence of a continuously differentiable solution) remain valid for discontinuous solutions with bounded variation, which are considered in the rest of the paper. This can be deemed as an extreme case of the Lavrentiev phenomenon (see [14]): In one Banach space there is a solution, and in another there are none. In [15] there appears the following remark: “Lavrentiev’s phenomenon is related to the existence of singular minimizers, i.e., absolutely continuous minimizers that are not Lipschitz.” In the case discussed in this work, the maximizers are of bounded variation but discontinuous, hence we can expect a Lavrentiev phenomenon. However, the optimal value can be approximated by a suitably chosen Lipschitz continuous function when *α* > 0, and probably not when *α* < 0. The functional (7) does not satisfy the coercivity condition (A4) from [14], especially not on any space that imposes restrictions on derivatives (of satiation), for the functional (8) does not even contain the derivative $\frac{ds}{dt}$ explicitly. This suggests that the result on the absence of a Lavrentiev gap in [14] can be proved without the coercivity condition.

## 4 Functions of bounded variation

If a model has no solutions, as was shown for the satiation model in Section 3, it has to be modified—we can “regularize” it by introducing an artificial viscosity or limiting the analysis to bounded consumption, as in [5],—or it could be abandoned in favor of another model. Alternatively, the definition of a solution could be changed. Intuition would suggest that the satiation model does have a solution and, as a result, an artificial regularization is not necessary. One alternative would be to relax the requirement that the satiation level *s*(*t*) be continuous, and instead allow *s* to have some points of discontinuity. If such discontinuities are permitted, the satiation *s*(*t*) is not differentiable in the classical sense at the points of discontinuity. However, *s*(*t*) is present in differential Eq (2), hence we need to use non-classical derivatives. In order to accommodate this relaxation, we permit *s* to be a function of bounded variation (BV); see [16] or [17]. In general, functions belonging to the BV space could be discontinuous, and their derivatives could be Borel measures. The Heaviside function, which is defined by *h*(*x*) = 0 for *x* ≤ 0 and *h*(*x*) = 1 for *x* > 0, is an example of a BV function. Its derivative is the well-known Borel measure popularly known as the Dirac delta function. For this reason, when satiation *s*(*t*) is a BV function, Eq (2) forces consumption *c* to be a Borel measure. Whenever satiation *s*(*t*) has a discontinuity, the singular part of the Borel measure *c* is the Dirac delta measure. In order to avoid this non-descriptive term, we use a more intuitive term for it: consumption gulp, following [4]. The mathematical description of a “gulp” or a gulp at a point of discontinuity of a function is given in Eq (25).

Functions of a single variable with bounded variation (BV functions) are continuous almost everywhere, and their derivatives are finite Borel measures. The three most important properties of BV functions are as follows:

1. The one-sided limits *φ*(*τ*^−^) = lim_*t*→*τ*,*t*<*τ*_ *φ*(*t*) and *φ*(*τ*^+^) = lim_*t*→*τ*,*t*>*τ*_ *φ*(*t*) exist whenever *τ* is in the closure of the interior of the domain of *φ*.
2. The derivatives exist even if the functions are discontinuous. The downside is that the derivatives are permitted to be Borel measures.
3. Integration by parts is permitted. If *φ* is a BV function on [*a*, *b*] and *g* is *C*^1^ on *R*, then $\frac{d\varphi }{dt}$ is a Borel measure and, if *φ*(*a*) and *φ*(*b*) are defined, we can integrate by parts as follows:
  $$\begin{array}{c} \int_{\left[a,b\right]}\varphi (t)\cdot \frac{dg}{dt}(t)\cdot dt=\varphi (b)\cdot g(b)-\varphi (a)\cdot g(a)-\int_{\left[a,b\right]}g(t)\cdot \frac{d\varphi }{dt}(t)\cdot dt \end{array}$$ (23)

If *φ* is *C*^1^ on *R* except for some point *τ* ∈ (*a*, *b*) and the limits *φ*(*τ*^+^) and *φ*(*τ*^−^) exist, then with the help of simple calculus we can proceed as follows:

$$\begin{array}{ccc} \int_{\left[a,b\right]}\varphi (t)\cdot \frac{dg}{dt}(t)\cdot dt & = & \int_{\left(a,\tau \right)}\varphi (t)\cdot \frac{dg}{dt}(t)\cdot dt+\int_{\left(\tau ,b\right)}\varphi (t)\cdot \frac{dg}{dt}(t)\cdot dt\\ & = & \varphi (b)\cdot g(b)-\varphi (a)\cdot g(a)-(\varphi (\tau^{+})-\varphi (\tau^{-}))\cdot g(\tau )\\ & & -\int_{\left(a,\tau \right)}g(t)\cdot \frac{d\varphi }{dt}(t)\cdot dt-\int_{\left(\tau ,b\right)}g(t)\cdot \frac{d\varphi }{dt}(t)\cdot dt \end{array}$$ (24)

In Eq (24), $\frac{d\varphi }{dt}$ denotes the classical derivative, which is defined everywhere except at *τ*. By comparing (23) to (24), we conclude that the Borel measure $\frac{d\varphi }{dt}$ is given by

$$\begin{array}{c} {\left[\frac{d\varphi }{dt}\right]}_{\text{generalized}}={\left[\frac{d\varphi }{dt}\right]}_{\text{classical}}+(\varphi (\tau^{+})-\varphi (\tau^{-}))\cdot \delta_{\tau }(t), \end{array}$$ (25)

where ${\left[\frac{d\varphi }{dt}(t)\right]}_{\text{classical}}$ is the classical derivative and (*φ*(*τ*^+^) − *φ*(*τ*^−^)) ⋅ *δ*_*τ*_(*t*) is the Dirac delta Borel measure with strength equal to the jump (*φ*(*τ*^+^) − *φ*(*τ*^−^)) in the value of *φ* at the discontinuity located at *t* = *τ*. This can be interpreted as a decomposition of the Borel measure $\frac{d\varphi }{dt}$ into the sum of the part that is continuous with respect to Lebesgue measure, ${\left[\frac{d\varphi }{dt}(t)\right]}_{\text{classical}}$, and the part that is singular with respect to Lebesgue measure, ${\left[\frac{d\varphi }{dt}\right]}_{\text{singular}}=(\varphi (\tau^{+})-\varphi (\tau^{-}))\cdot \delta_{\tau }(t)$. As mentioned earlier, in this paper the latter is referred to as a “gulp.” The theory of BV functions provides us with derivatives of discontinuous functions and formulas for integration by parts which are applicable to discontinuous functions, even if the singular parts of their derivatives are much more complicated than in the example above (e.g., the singular part of the derivative of the Cantor function).

The use of integration by parts to derive formulas (18) and (13), as well as formula (69) in Section 7.3, is justified by the formula in (23) when *s*(*t*) is a BV function. Hence all of the identities involving integrals derived in the preceding sections are also valid when *C*^1^ differentiability is relaxed to that in the BV sense. In this paper the discontinuous function is satiation *s*(*t*). If *s*(*t*) is discontinuous at *t* = *T*, then Eq (2) implies that this contributes $\frac{s(T)-s(T^{-})}{f}\cdot \delta_{T}(t)$ to consumption. If we know that the discontinuities in satiation can occur only at the endpoints of the interval [0, *T*], we can avoid BV theory altogether, as was done in [4]. However, in order to prove this, one needs to initially permit the derivative of the satiation to be a general Borel measure on [0, *T*] and then to show that it is continuous with respect to Lebesgue measure in (0, *T*). Also, a discontinuity of *s*(*t*) at *t* = *T* allows us to remove the contradiction obtained in the proof of Proposition 8. This is the approach used in the proof of Proposition 14 in the next section.

**Remark 9** Eq (23) *for open sets takes the form*

$$\begin{array}{c} \int_{\left(a,b\right)}\varphi (t)\cdot \frac{dg}{dt}(t)\cdot dt=\varphi (b^{-})\cdot g(b)-\varphi (a^{+})\cdot g(a)-\int_{\left(a,b\right)}g(t)\cdot \frac{d\varphi }{dt}(t)\cdot dt. \end{array}$$ (26)

*Eqs* (26) *and* (23) *imply that*

$$\begin{array}{ccc} \int_{\left[a,b\right]}g(t)\cdot \frac{d\varphi }{dt}(t)\cdot dt & = & \int_{\left(a,b\right)}g(t)\cdot \frac{d\varphi }{dt}(t)\cdot dt\\ & & \;+g(b)\cdot (\varphi (b)-\varphi (b^{-}))+g(a)\cdot (\varphi (a^{-})-\varphi (a)). \end{array}$$

## 5 Sufficiency and necessity

In Section 3 it was shown that the space *C*^1^([0, *T*] → *R*) × *C*^1^ ([0, *T*] → *R*) × *R* is inadequate for investigation of solutions of Problem 7, hence we modify that problem as follows:

**Problem 10 (BV version)** *Find* [*s* (*t*), λ_*c*_ (*t*), λ_*W*_] ∈ *BV* ([0, *T*] → *R*) × *BV* ([0, *T*] → *R*) × *R that satisfies* (2), (20), *initial condition* (3), *terminal condition* (19), *constraints* (4) and (5), *constraint* (13) *with W* > 0, *and KKT conditions* (17).

In spite of discontinuities and exotic derivatives, the use of integration by parts is valid for the functions in *BV* ([0, *T*] → *R*), hence all the equations and conclusions obtained in Sections 2 and 3, including Eq (18) and the conditions (19) and (20), remain valid under the premises of Problem 10. Proposition 5 is also valid, and the proof is almost the same: When the consumption *c* is a non-negative measure, it replaces *c*(*t*_1_) ⋅ *dt*_1_, and the second term inside the parentheses in Eq (15) is still positive for all *t* ∈ (0, *T*].

In Section 2 it was proved that if [*s*, λ_*c*_, λ_*W*_] ∈ *C*^1^ ([0, *T*] → *R*) × *C*^1^ ([0, *T*] → *R*) × *R* is a stationary point of the Lagrangian functional in (16), then [*s*, λ_*c*_, λ_*W*_] must be a solution of Problem 7. With the help of the BV theory, one can easily prove sufficiency of conditions (19) and (20) in Problem 10.

**Theorem 11 (on sufficiency)** *Suppose V and V*_*S*_ *are concave-down, continuously differentiable functions, and let* [*s*, λ_*c*_, λ_*W*_] ∈ *BV* ([0, *T*] → *R*) × *BV* ([0, *T*] → *R*) × *R be a solution of Problem 10. Then the functional* (8) *attains a maximum value on the set*

$$\begin{array}{c} \left\{s_{1}\in BV([0,T]\to R)\;|\;s_{1}\;satisfies\;all\;the\;constraints\;of\;Problem\;10\right\} \end{array}$$ (27)

*at s*.

**Proof**. Let Δ*s* ∈ *BV* ([0, *T*] → *R*) be any function such that *s* + Δ*s* satisfies (2), initial condition (3), constraint (4), constraint (13) with *W* > 0, and (5). Then for all *x* ∈ [0, 1] the function *s*_*x*_ = (1 − *x*) ⋅ *s* + *x* ⋅ (*s* + Δ*s*) = *s* + *x* ⋅ Δ*s* also satisfies these conditions. Consider the function

$$\begin{array}{c} \Theta (x)=\varphi \cdot U_{S}(s_{x}(t))=e^{-n\cdot T}\cdot V(s_{x}(T))-V(s_{0})+\int_{\left[0,T\right]}e^{-n\cdot t}\cdot V_{S}(s_{x}(t))\cdot dt, \end{array}$$

where *U*_*S*_ is given by (8). Since *s* and Δ*s* are bounded, the bounded convergence theorem ensures that Θ is differentiable with respect to *x* and

$$\begin{array}{ccc} {\left[\frac{d\Theta }{dx}\right]}_{x=0} & = & \varphi \cdot {\left[\frac{dU_{S}(s_{x}(t))}{dx}\right]}_{x=0}\\ & = & {\left[\frac{d}{dx}(e^{-n\cdot T}\cdot V((s+x\cdot \Delta s)(T))-V(s_{0}))\right]}_{x=0}\\ & & +{\left[\frac{d}{dx}(\int_{\left[0,T\right]}e^{-n\cdot t}\cdot V_{S}((s+x\cdot \Delta s)(t))\cdot dt)\right]}_{x=0}\\ & = & e^{-n\cdot T}\cdot \frac{dV}{ds}(s(T))\cdot \Delta s(T)\\ & & +\int_{\left[0,T\right]}e^{-n\cdot t}\cdot \frac{dV_{S}}{ds}(s(t))\cdot \Delta s(t)\cdot dt \end{array}$$

Since [*s*, λ_*c*_, λ_*W*_] is a solution of Problem 10, we may use (20) and (19), and we obtain

$$\begin{array}{ccc} {\left[\frac{d\Theta }{dx}\right]}_{x=0} & = & \left[\lambda_{W}\cdot e^{-r\cdot T}-\lambda_{c}(T)]\cdot \Delta s(T\right)\\ & & +\int_{\left[0,T\right]}[\lambda_{W}\cdot e^{-r\cdot t}\cdot (\gamma +r)+\frac{d\lambda_{c}}{dt}-\lambda_{c}\cdot \gamma ]\cdot \Delta s(t)\cdot dt\\ & = & \lambda_{W}\cdot e^{-r\cdot T}\cdot \Delta s(T)+\int_{\left[0,T\right]}\lambda_{W}\cdot e^{-r\cdot t}\cdot (\gamma +r)\cdot \Delta s(t)\cdot \\ & & -\lambda_{c}(T)\cdot \Delta s(T)+\int_{\left[0,T\right]}[\frac{d\lambda_{c}}{dt}-\lambda_{c}\cdot \gamma ]\cdot \Delta s(t)\cdot dt \end{array}$$

Now constraint (13) implies that

$$\begin{array}{ccc} & & \lambda_{W}\cdot e^{-r\cdot T}\cdot \Delta s(T)+\int_{\left[0,T\right]}\lambda_{W}\cdot e^{-r\cdot t}\cdot (\gamma +r)\cdot \Delta s(t)\cdot dt\\ & = & \frac{d}{dx}(\lambda_{W}\cdot e^{-r\cdot T}\cdot s_{x}(T)+\lambda_{W}\cdot \int_{\left[0,T\right]}e^{-r\cdot t}\cdot (\gamma +r)\cdot s_{x}(t)\cdot dt)\\ & = & \frac{d}{dx}(\varphi \cdot W)=0, \end{array}$$

so

$$\begin{array}{c} {\left[\frac{d\Theta }{dx}\right]}_{x=0}=-\lambda_{c}(T)\cdot \Delta s(T)+\int_{\left[0,T\right]}[\frac{d\lambda_{c}}{dt}-\lambda_{c}\cdot \gamma ]\cdot \Delta s(t)\cdot dt \end{array}$$

Integration by parts produces

$$\begin{array}{c} \int_{\left[0,T\right]}\frac{d\lambda_{c}}{dt}\cdot \Delta s(t)\cdot dt=\lambda_{c}(T)\cdot \Delta s(T)-\int_{\left[0,T\right]}\frac{d\Delta s}{dt}\cdot \lambda_{c}(t)\cdot dt \end{array}$$

since Δ*s*(0) = 0, hence

$$\begin{array}{ccc} {\left[\frac{d\Theta }{dx}\right]}_{x=0} & = & -\int_{\left[0,T\right]}[\frac{d\Delta s}{dt}+\Delta s\cdot \gamma ]\cdot \lambda_{c}(t)\cdot dt\\ & = & -\int_{\text{supp}(\lambda_{c})}[\frac{d\Delta s}{dt}+\Delta s\cdot \gamma ]\cdot \lambda_{c}(t)\cdot dt. \end{array}$$

Now note the following: λ_*c*_(*t*) ≥ 0 and *c*(*t*) = 0 when *t* ∈ supp(λ_*c*_), thanks to KKT conditions (17). Therefore, $[\frac{d\Delta s}{dt}+\Delta s\cdot \gamma ](t)\geq 0$ when *t* ∈ supp(λ_*c*_), since *s* + Δ*s* satisfies (5). Hence $[\frac{d\Delta s}{dt}+\Delta s\cdot \gamma ]\cdot \lambda_{c}(t)\geq 0$ on supp(λ_*c*_), and we conclude that $\frac{d\Theta }{dx}(0)\leq 0$. This implies that Θ(*x*) attains a maximum at *x* = 0, that is, at *s*, since the function Θ is concave down on [0, 1]. But the functional *BV* ([0, *T*] → *R*) ∋ *s* → *U*_*S*_(*s*) is continuous on *BV* ([0, *T*] → *R*), thanks to continuity of *V*_*S*_, and this ensures that *s* is also a maximum of the convex functional *U*_*S*_(*s*) on the set in (27). Thus *s* is unique, thanks to the downward concavity of *U*_*S*_.

**Remark 12** *A proof of necessity of the conditions in Problem 10 is not needed for explicit construction of solutions in the sections of the paper that follow*. *For the sake of completeness, however, we present a proof of the necessity*.

**Theorem 13 (on necessity)** *Let V be a twice continuously differentiable function, let c be a non-negative Borel measure on* [0, *T*] *that satisfies* (6), *and let s be the solution of the differential* Eq (2) *that satisfies* (3) *and* (4). *Then s* ∈ *BV* ([0, *T*] → *R*). *Suppose* [*c*, *s*] *maximizes the functional* (8) *under constraints* (5), (6) *with W* > 0, (2), (3), *and* (4). *Then there exist a non-negative Lipschitz continuous function* λ_*c*_ ∈ *Lip* ([0, *T*] → *R*) *and a non-negative number* λ_*W*_ ∈ *R such that* [*s*, λ_*c*_, λ_*W*_] ∈ *BV*([0, *T*] → *R*) × *Lip* ([0, *T*] → *R*) × *R is a solution of Problem (10)*.

**Proof**. If *c* is a Borel measure, then the solution of the initial-value problem (Problem 2) with the initial condition (3) is given by (15), hence the solution is bounded, which means that $\frac{ds}{dt}$ is a Borel measure thanks to (2), and so *s* ∈ *BV* ([0, *T*] → *R*). Suppose that under the constraints listed in the Theorem, the functional (8) attains its maximum value on *BV* ([0, *T*] → *R*) at *s*. We need to construct λ_*c*_ ∈ *Lip* ([0, *T*] → *R*) and a λ_*W*_ ∈ *R*, and show that λ_*c*_ ≥ 0 and λ_*W*_ > 0, and that [*s*, λ_*c*_, λ_*W*_] satisfy the differential Eq (20) and the terminal condition (19). Let *c*_1_ be any non-negative Borel measure on [0, *T*] that satisfies (6), and let *s*_1_ be the corresponding solution of the initial-value problem that consists of Eq (2) together with (3) and (4). In addition, for all *x* ∈ [0, 1], let *c*_*x*_ = (1 − *x*) ⋅ *c* + *x* ⋅ *c*_1_ and *s*_*x*_ = (1 − *x*) ⋅ *s* + *x* ⋅ *s*_1_. Then *c*_*x*_ is non-negative and also satisfies (6), and *s*_*x*_ ∈ *BV* ([0, *T*] → *R*). Consider the function of *x* ∈ [0, 1] given by

$$\begin{array}{ccc} U_{S}(s_{x},c_{x}) & = & \int_{\left[0,T\right]}e^{-n\cdot t}\cdot \frac{dV}{ds}(s_{x}(t))\cdot c_{x}(t)\cdot dt\\ & = & (1-x)\cdot \int_{\left[0,T\right]}e^{-n\cdot t}\cdot \frac{dV}{ds}(s_{x}(t))\cdot c(t)\cdot dt\\ & & +\;x\cdot \int_{\left[0,T\right]}e^{-n\cdot t}\cdot \frac{dV}{ds}(s_{x}(t))\cdot c_{1}(t)\cdot dt, \end{array}$$ (28)

where the integrals above are Lebesgue-type integrals with respect to Borel measures. Since *s* and *s*_1_ are both bounded, with the help of the bounded convergence theorem one can prove that *U*_*S*_(*s*_*x*_, *c*_*x*_) is differentiable with respect to *x* and that

$$\begin{array}{ccc} \frac{d}{dx}U_{S}(s_{x},c_{x}) & = & \frac{d}{dx}\int_{\left[0,T\right]}e^{-n\cdot t}\cdot \frac{dV}{ds}(s_{x}(t))\cdot c_{x}(t)\cdot dt\\ & = & \int_{\left[0,T\right]}e^{-n\cdot t}\cdot \frac{dV}{ds}(s(t))\cdot c_{1}(t)\cdot dt\\ & & -\int_{\left[0,T\right]}e^{-n\cdot t}\cdot \frac{dV}{ds}(s(t))\cdot c(t)\cdot dt\\ & & +(1-x)\cdot \int_{\left[0,T\right]}e^{-n\cdot t}\cdot \frac{d^{2}V}{ds^{2}}(s_{x}(t))\cdot (s_{1}-s)\cdot c(t)\cdot dt\\ & & +x\cdot \int_{\left[0,T\right]}e^{-n\cdot t}\cdot \frac{d^{2}V}{ds^{2}}(s_{x}(t))\cdot (s_{1}-s)\cdot c_{1}(t)\cdot dt \end{array}$$

Since *U*_*S*_(*s*_*x*_, *c*_*x*_) attains its maximum at *x* = 0, we have

$$\begin{array}{ccc} 0 & \geq & {\left(\frac{d}{dx}U_{S}(s_{x},c_{x})\right)}_{x=0}\\ & = & \int_{\left[0,T\right]}e^{-n\cdot t}\cdot \frac{dV}{ds}(s_{x}(t))\cdot (c_{1}(t)-c(t))\cdot dt\\ & & +\int_{\left[0,T\right]}e^{-n\cdot t}\cdot \frac{d^{2}V}{ds^{2}}(s(t))\cdot (s_{1}-s)(t)\cdot c(t)\cdot dt \end{array}$$

The second integral may be transformed as follows:

$$\begin{array}{ccc} & & \int_{\left[0,T\right]}e^{-n\cdot t}\cdot \frac{d^{2}V}{ds^{2}}(s(t))\cdot (s_{1}-s)(t)\cdot c(t)\cdot dt\\ & & \text{(with}\;\text{the}\;\text{help}\;\text{of}\;(2))\\ & = & \int_{\left[0,T\right]}e^{-n\cdot t}\cdot \frac{d^{2}V}{ds^{2}}(s(t))\cdot (s_{1}-s)(t)\cdot \frac{1}{\varphi }\cdot (\frac{ds}{dt}+\gamma \cdot s)\cdot dt\\ & = & \frac{1}{\varphi }\cdot \int_{\left[0,T\right]}e^{-n\cdot t}\cdot (s_{1}-s)(t)\cdot (\frac{d}{dt}(\frac{dV}{ds}(s(t)))+\gamma \cdot s\cdot \frac{d^{2}V}{ds^{2}}(s(t)))\cdot dt \end{array}$$

$$\begin{array}{ccc} & & (\;\text{integration}\;\text{by}\;\text{parts)}\\ & = & \frac{1}{\varphi }\cdot e^{-n\cdot T}\cdot V(s(T))\cdot (s_{1}-s)(T)\\ & & +\frac{1}{\varphi }\cdot \int_{\left[0,T\right]}e^{-n\cdot t}\cdot (n\cdot \frac{dV}{ds}(s(t))+\gamma \cdot s\cdot \frac{d^{2}V}{ds^{2}}(s(t)))\cdot (s_{1}-s)(t)\cdot dt\\ & & -\frac{1}{\varphi }\cdot \int_{\left[0,T\right]}e^{-n\cdot t}\cdot \frac{dV}{ds}(s(t))\cdot \frac{d}{dt}(s_{1}-s)(t)\cdot dt\\ & & \text{(since}\;s\;\text{and}\;s_{1}\;\text{satisfy}\;(2)\;\text{with}\;c\;\text{and}\;c_{1}\text{,}\;\text{respectively)} \end{array}$$

$$\begin{array}{ccc} & = & \frac{1}{\varphi }\cdot e^{-n\cdot T}\cdot \frac{dV}{ds}(s(T))\cdot (s_{1}-s)(T)\\ & & +\frac{1}{\varphi }\cdot \int_{\left[0,T\right]}e^{-n\cdot t}\cdot (n\cdot \frac{dV}{ds}(s(t))+\gamma \cdot s\cdot \frac{d^{2}V}{ds^{2}}(s(t))\\ & & \;\;\;\;\;\;\;\;\;\;\;\;\;\;\;\;\;\;\;\;\;\;\;\;\;\;\;\;\;\;\;\;\;+\gamma \cdot \frac{dV}{ds}(s(t)))\cdot (s_{1}-s)(t)\cdot dt\\ & & -\int_{\left[0,T\right]}e^{-n\cdot t}\cdot \frac{dV}{ds}(s(t))\cdot (c_{1}-c)(t)\cdot dt, \end{array}$$

hence thanks to (10),

$$\begin{array}{ccc} 0 & \geq & {\left(\frac{d}{dx}U_{S}(s_{x},c_{x})\right)}_{x=0}\\ & = & \frac{1}{\varphi }\cdot e^{-n\cdot T}\cdot \frac{dV}{ds}(s(T))\cdot (s_{1}-s)(T)\\ & & +\frac{1}{\varphi }\cdot \int_{\left[0,T\right]}e^{-n\cdot t}\cdot \frac{dV_{S}}{ds}\cdot (s_{1}-s)(t)\cdot dt. \end{array}$$

Since

$$\begin{array}{c} (s_{1}-s)(t)=e^{-\gamma \cdot t}\cdot \varphi \cdot \int_{\left[0,T\right]}e^{\gamma \cdot t_{1}}\cdot (c_{1}(t_{1})-c(t_{1}))\cdot dt_{1}, \end{array}$$

we have

$$\begin{array}{ccc} 0 & \geq & {\left(\frac{d}{dx}U_{S}(s_{x},c_{x})\right)}_{x=0}\\ & = & \frac{1}{\varphi }\cdot e^{-n\cdot T}\cdot \frac{dV}{ds}(s(T))\cdot e^{-\gamma \cdot T}\cdot \varphi \cdot \int_{\left[0,T\right]}e^{\gamma \cdot t}\cdot (c_{1}(t)-c(t))\cdot dt\\ & & +\frac{1}{\varphi }\cdot \int_{\left[0,T\right]}e^{-n\cdot t}\cdot \frac{dV_{S}}{ds}\cdot e^{-\gamma \cdot t}\cdot \varphi \cdot \int_{\left[0,T\right]}e^{\gamma \cdot t_{1}}\cdot (c_{1}(t_{1})-c(t_{1}))\cdot dt_{1}\cdot dt \end{array}$$

Thanks to Fubini’s theorem, ∫_[0,*T*]_ ∫_[0,*t*]_… ⋅ *dt*_1_ ⋅ *dt* = ∫_[0,*T*]_ ∫_[*t*, *T*]_… ⋅ *dt* ⋅ *dt*_1_, hence

$$\begin{array}{cc} & \frac{1}{\varphi }\cdot \int_{\left[0,T\right]}e^{-n\cdot t}\cdot \frac{dV_{S}}{ds}\cdot e^{-\gamma \cdot t}\cdot \varphi \cdot \int_{\left[0,T\right]}e^{\gamma \cdot t_{1}}\cdot (c_{1}(t_{1})-c(t_{1}))\cdot dt_{1}\cdot dt\\ = & \int_{\left[0,T\right]}[\int_{\left[t,T\right]}e^{-(n+\gamma )\cdot t_{1}}\cdot \frac{dV_{S}}{ds}(s(t_{1}))\cdot dt_{1}\cdot e^{\gamma \cdot t}\cdot (c_{1}(t)-c(t))]\cdot dt, \end{array}$$

so

$$\begin{array}{ccc} 0 & \geq & {\left(\frac{d}{dx}U_{S}(s_{x},c_{x})\right)}_{x=0}\\ & = & \frac{1}{\varphi }\cdot e^{-n\cdot T}\cdot \frac{dV}{ds}(s(T))\cdot e^{-\gamma \cdot T}\cdot \varphi \cdot \int_{\left[0,T\right]}e^{\gamma \cdot t}\cdot (c_{1}(t)-c(t))\cdot dt\\ & & +\frac{1}{\varphi }\cdot \int_{\left[0,T\right]}e^{-n\cdot t}\cdot \frac{dV_{S}}{ds}\cdot e^{-\gamma \cdot t}\cdot \varphi \cdot \int_{\left[0,T\right]}e^{\gamma \cdot t_{1}}\cdot (c_{1}(t_{1})-c(t_{1}))\cdot dt_{1}\cdot dt \end{array}$$

$$\begin{array}{ccc} & = & \frac{dV}{ds}(s(T))\cdot e^{-(n+\gamma )\cdot T}\cdot \int_{\left[0,T\right]}e^{\gamma \cdot t}\cdot (c_{1}(t)-c(t))\cdot dt\\ & & +\int_{\left[0,T\right]}\int_{\left[t,T\right]}e^{-(n+\gamma )\cdot t_{1}}\cdot \frac{dV_{S}}{ds}(s(t_{1}))\cdot dt_{1}\cdot e^{\gamma \cdot t}\cdot (c_{1}(t)-c(t))\cdot dt\\ & = & \int_{\left[0,T\right]}M(t)\cdot (c_{1}-c)(t)\cdot dt, \end{array}$$

where

$$\begin{array}{c} M(t)=e^{\gamma \cdot t}\cdot (\frac{dV}{ds}(s(T))\cdot e^{-(n+\gamma )\cdot T}+\int_{\left[t,T\right]}e^{-(n+\gamma )\cdot t_{1}}\cdot \frac{dV_{S}}{ds}(s(t_{1}))\cdot dt_{1})>0 \end{array}$$ (29)

since $\frac{dV_{S}}{ds}>0$ and $\frac{dV}{ds}>0$. Hence we obtain

$$\begin{array}{c} \int_{\left[0,T\right]}M(t)\cdot c(t)\cdot dt\geq \int_{\left[0,T\right]}M(t)\cdot c_{1}(t)\cdot dt, \end{array}$$

which means that the Borel measure *c* maximizes ∫_[0,*T*]_ *M*(*t*) ⋅ *c*_1_(*t*) ⋅ *dt* on the set of all non-negative Borel measures *c*_1_ that satisfy constraint (6). Since *M* > 0, the intuition behind the solution of this problem is as follows: Let

$$\begin{array}{c} \lambda_{W}=\underset{t\in [0,T]}{\sup}(M(t)\cdot e^{r\cdot t})>0 \end{array}$$

Then the optimal *c* is any non-negative Borel measure that satisfies (6) and

$$\begin{array}{c} \text{supp}\;(c)\subset \{t\in [0,T]\;|\;M(t)\cdot e^{r\cdot t}=\lambda_{W}\}, \end{array}$$

in which case

$$\begin{array}{ccc} \int_{\left[0,T\right]}M(t)\cdot c(t)\cdot dt & = & \int_{\text{supp}(c)}M(t)\cdot c(t)\cdot dt\\ & = & \int_{\text{supp}(c)}M(t)\cdot e^{r\cdot t}\cdot e^{-r\cdot t}\cdot c(t)\cdot dt\\ & = & \lambda_{W}\cdot \int_{\text{supp}(c)}e^{-r\cdot t}\cdot c(t)\cdot dt=\lambda_{W}\cdot W \end{array}$$

If *c*_1_ is any non-negative Borel measure on [0, *T*] that satisfies (6), then

$$\begin{array}{ccc} \int_{\left[0,T\right]}M(t)\cdot c_{1}(t)\cdot dt & = & \int_{\left[0,T\right]}e^{r\cdot t}\cdot M(t)\cdot e^{-r\cdot t}\cdot c_{1}(t)\cdot dt\\ & \leq & \lambda_{W}\cdot \int_{\left[0,T\right]}e^{-r\cdot t}\cdot c_{1}(t)\cdot dt=\lambda_{W}\cdot W\\ & = & \int_{\left[0,T\right]}M(t)\cdot c(t)\cdot dt \end{array}$$

Thus *c* maximizes ∫_[0,*T*]_ *M*(*t*) ⋅ *c*_1_(*t*) ⋅ *dt* on the set of all non-negative Borel measures *c*_1_ on [0, *T*] that satisfy (6). Now let

$$\begin{array}{c} \lambda_{c}(t)=\lambda_{W}\cdot e^{-r\cdot t}-M(t) \end{array}$$ (30)

Obviously, λ_*c*_ ≥ 0 and λ_*c*_(*t*) = 0 for *t* ∈ supp(*c*), hence λ_*c*_ satisfies KKT condition (17). Next, (2), (3), (4), and (5) imply that *s* > 0 on the compact interval [0, *T*], hence inf_*t*∈[0,*T*]_ (*s*(*t*)) > 0. Thanks to $\frac{d^{2}V_{S}}{ds^{2}}<0$ (which is equivalent to *α*_*S*_ > 0), this implies that $\frac{dV_{S}}{ds}(s)$ is bounded on [0, *T*], which in turn implies that *M* ∈ *Lip* ([0, *T*] → *R*), where *M* is given by (29). Thus λ_*c*_ ∈ *Lip* ([0, *T*] → *R*), where λ_*c*_ is given by (30). We need to show that λ_*c*_ satisfies the boundary condition (19) and the differential Eq (20). Indeed,

$$\begin{array}{c} M(T)=e^{\gamma \cdot T}\cdot \frac{dV}{ds}(s(T))\cdot e^{-(n+\gamma )\cdot T}=\frac{dV}{ds}(s(T))\cdot e^{-n\cdot T}, \end{array}$$

hence

$$\begin{array}{c} \lambda_{c}(T)=\lambda_{W}\cdot e^{-r\cdot T}-M(T)=\lambda_{W}\cdot e^{-r\cdot T}-\frac{dV}{ds}(s(T))\cdot e^{-n\cdot T}, \end{array}$$

so λ_*c*_ satisfies the boundary condition (19). Next,

$$\begin{array}{ccc} \frac{d}{dt}M(t) & = & \frac{d}{dt}[e^{\gamma \cdot t}\cdot (\frac{dV}{ds}(s(T))\cdot e^{-(n+\gamma )\cdot T}\\ & & \;\;\;\;\;\;\;\;\;\;\;\;\;\;\;\;\;\;\;\;\;\;\;\;\;\;\;\;\;+\int_{\left[t,T\right]}e^{-(n+\gamma )\cdot t_{1}}\cdot \frac{dV_{S}}{ds}(s(t_{1}))\cdot dt_{1})]\\ & = & \gamma \cdot M+e^{\gamma \cdot t}\cdot \frac{d}{dt}(\frac{dV}{ds}(s(T))\cdot e^{-(n+\gamma )\cdot T}\\ & & \;\;\;\;\;\;\;\;\;\;\;\;\;\;\;\;\;\;\;\;\;\;\;\;\;\;\;\;\;+\int_{\left[t,T\right]}e^{-(n+\gamma )\cdot t_{1}}\cdot \frac{dV_{S}}{ds}(s(t_{1}))\cdot dt_{1})\\ & = & \gamma \cdot M+e^{\gamma \cdot t}\cdot \frac{d}{dt}\int_{\left[t,T\right]}e^{-(n+\gamma )\cdot t_{1}}\cdot \frac{dV_{S}}{ds}(s(t_{1}))\cdot dt_{1}\\ & = & \gamma \cdot M-e^{\gamma \cdot t}\cdot e^{-(n+\gamma )\cdot t}\cdot \frac{dV_{S}}{ds}(s(t))=\gamma \cdot M-e^{-n\cdot t}\cdot \frac{dV_{S}}{ds}(s(t)), \end{array}$$

so

$$\begin{array}{ccc} \frac{d}{dt}\lambda_{c}(t)-\lambda_{c}\cdot \gamma & = & \frac{d}{dt}(\lambda_{W}\cdot e^{-r\cdot t}-M(t))-(\lambda_{W}\cdot e^{-r\cdot t}-M(t))\cdot \gamma \\ & = & -(r+\gamma )\cdot \lambda_{W}\cdot e^{-r\cdot t}-\frac{d}{dt}M(t)+M(t)\cdot \gamma \\ & = & -(r+\gamma )\cdot \lambda_{W}\cdot e^{-r\cdot t}+e^{-n\cdot t}\cdot \frac{dV_{S}}{ds}(s(t)), \end{array}$$

which is the differential Eq (20).

The two proofs above use ideas from [18]. A few minor gaps in that material are covered here.

## 6 Structure of the optimal solutions

In this section the structure of solutions of Problem (10) is investigated, the main results being Theorems (21) and (25).

Discontinuities complicate the proofs, but they do not destroy the conclusions of Section 2. To a large extent, functions of bounded variation that are not continuously differentiable can be treated formally as if they were (e.g., in terms of differentiation and integration by parts). Note also that condition (5) and Eq (25) require the gulp of consumption $c\left(t\right)=\frac{s\left(t^{+}\right)-s\left(t^{-}\right)}{\varphi }$ at each jump in the satiation to be positive, that is, they require the singular part of *c* to be positive as well as the part that is continuous with respect to Lebesgue measure.

We first show that a discontinuity in the satiation at *t* = *T* is essential, as it allows one to resolve the contradiction that arose in the proof of Proposition (8). When functions with bounded variation are considered, Eq (20) reduces to

$$\begin{array}{c} 0=e^{-n\cdot T}\cdot \frac{dV_{S}}{ds}(s(T^{-}))-\lambda_{W}\cdot e^{-r\cdot T}\cdot (\gamma +r), \end{array}$$ (31)

where *s*(*T*^−^) = lim_*t*→*T*,*t*<*T*_ *s*(*t*). Eq (31) replaces Eq (22). If there is a discontinuity at *t* = *T*, then *s*(*T*^−^) ≠ *s*(*T*); this provides an extra degree of freedom, which removes the contradiction between Eqs (21) and (31) at *t* = *T* that eliminated continuous solutions. We can prove even more:

**Proposition 14** *Let* [*s*, λ_*c*_, λ_*W*_] ∈ *BV* ([0, *T*] → *R*) × *BV* ([0, *T*] → *R*) × *R be a solution of Problem 10*. *If* $\frac{dV}{ds}(s)>0$ *and* $\frac{d^{2}V}{ds^{2}}(s)<0$ *for all s, then the following hold*:

1. *There must be a gulp of consumption at t* = *T if for all s*
  $$\begin{array}{c} n<r+\alpha \cdot \gamma . \end{array}$$ (32)
2. *A gulp of consumption at t* = *T is impossible if for all s*
  $$\begin{array}{c} n>r+\alpha \cdot \gamma . \end{array}$$ (33)

**Proof**. When λ_*W*_ is eliminated between (21) and (31) for *t* = *T*, one obtains

$$\begin{array}{c} 0=e^{-n\cdot T}\cdot (\frac{dV_{S}}{ds}(s(T^{-}))-\frac{dV}{ds}(s(T))\cdot (\gamma +r)), \end{array}$$

where *s*(*T*^−^) = lim_*t*→*T*,*t*<*T*_ *s*(*t*). With the help of (10), this can be transformed into

$$\begin{array}{c} \frac{dV}{ds}(s(T))=\frac{dV_{S}}{ds}(s(T^{-}))\cdot \frac{1}{\left(\gamma +r\right)}=\frac{dV}{ds}(s(T^{-}))\cdot (1-\frac{r+\gamma \cdot \alpha -n}{\left(\gamma +r\right)}), \end{array}$$ (34)

hence $\frac{dV}{ds}(s(T))-\frac{dV}{ds}(s(T^{-}))=\frac{dV}{ds}(s(T^{-}))\cdot \frac{n-r-\alpha \cdot \gamma }{\left(\gamma +r\right)}$. Thus there is a discontinuity, *s*(*T*) ≠ *s*(*T*^−^), if *n* − *r* − *α*(*s*) ⋅ *γ* ≠ 0 for all *s*.

If (32) holds, then $\frac{dV}{ds}(s(T))-\frac{dV}{ds}(s(T^{-}))<0$, hence *s*(*T*) − *s*(*T*^−^) > 0 because $\frac{d^{2}V}{ds^{2}}(s)<0$.

This discontinuity of *s*(*t*) produces the singular part of $\frac{ds}{dt}{|}_{t=T}=s(T)-s(T^{-})$; see Section 4. Now Eq (2) implies that the consumption *c* has singular part $\frac{s(T)-s(T^{-})}{f}$, which we call a consumption gulp. This gulp must be positive, since consumption *c* must be positive; see (5). However, (33) implies that the gulp of consumption must be negative. Therefore, a gulp of consumption at *T* is impossible if (33) holds.

Proposition 14 resolves the non-existence contradiction from Section 3: The optimal solutions must be discontinuous at *t* = *T* if (32) holds for all *s*. The next lemma provides the first step in deciphering the structure of the optimal solutions.

**Lemma 15** *If* [*s*, λ_*c*_, λ_*W*_] ∈ *BV* ([0, *T*] → *R*) × *BV* ([0, *T*] → *R*) × *R is a solution of Problem 10*, *then* λ_*c*_(*t*) ∈ *Lip* ([0, *T*] → *R*).

**Proof**. The satiation *s* is bounded (although it may be discontinuous if *c* is not absolutely continuous with respect to Lebesgue measure), because every function of bounded variation is bounded; see [16]. The boundedness of *s* and condition (19) imply boundedness of λ_*c*_(*T*), which, together with (20) and the help of the Gronwall lemma, implies boundedness of λ_*c*_(*t*) on [0, *T*]. This property, together with (20), implies boundedness of $\frac{d\lambda_{c}(t)}{dt}$, hence λ_*c*_(*t*) is Lipschitz continuous.

Continuity of λ_*c*_ permits us to deduce the following result:

**Proposition 16** *Let* [*s*, λ_*c*_, λ_*W*_] ∈ *BV* ([0, *T*] → *R*) × *Lip* ([0, *T*] → *R*) × *R be a solution of Problem 10*. *Then there is a family of at most countably many (relatively) open subintervals of* [0, *T*] *such that* λ_*c*_ > 0 *and c* = 0 *in these subintervals, and* λ_*c*_ = 0 *and c* ≥ 0 *at the isolated points as well as on the closed subintervals that separate these open subintervals*.

**Proof**. Thanks to Lemma 15, λ_*c*_(*t*) is continuous, hence the inverse image of (0, +∞) is relatively open in [0, *T*]. Since the set of rational numbers, which is countable, is dense in the set of all real numbers, there is at most a countable set of (relatively) open subintervals of [0, *T*] in which λ_*c*_ > 0 and *c* = 0, while λ_*c*_ = 0 and *c* > 0 on each closed subinterval and at each single point that separates any two consecutive (relatively) open subintervals of [0, *T*] in which λ_*c*_ > 0.

In the remaining part of this section, we prove that there can be at most one (relatively) open subinterval in which λ_*c*_ > 0 and *c* = 0, and at most one closed subinterval on which λ_*c*_ = 0 and *c* > 0; see Theorems 21 and 25. In the next lemma, we construct a basic block of the explicit solution of Problem 10 in open subintervals where λ_*c*_ = 0 and *c* > 0. In this case, Eq (20) simplifies to

$$\begin{array}{c} \frac{e^{-(n-r)t}}{\left(\gamma +r\right)}\cdot \frac{dV_{S}}{ds}(\sigma )=\lambda \end{array}$$ (35)

with λ = λ_*W*_ ∈ *R*. Eq (35) does not contain $\frac{d\sigma }{dt}$, and this simplifies forthcoming proofs.

**Lemma 17** *If* $\frac{dV_{S}}{ds}(s)>0$ *and α*_*S*_(*s*) > 0 *for all s* ≥ 0, *and if both of the following hold*,

$$\begin{array}{ccc} \lim_{s\to 0^{+}}\frac{dV_{S}}{ds} & = & +\infty \end{array}$$ (36)

$$\begin{array}{ccc} \lim_{s\to \infty }\frac{dV_{S}}{ds} & = & 0, \end{array}$$ (37)

*then* Eq (35) *has a unique bounded, continuous positive solution for all t* ≥ 0 *and* 0 < λ ∈ *R that satisfy* Eq (39). *The corresponding optimal consumption is given by*

$$\begin{array}{c} c(t,\lambda )=\frac{r+\alpha_{S}\cdot \gamma -n}{f\cdot \alpha_{S}}\cdot \sigma (t,\lambda ) \end{array}$$ (38)

**Proof**. The conditions $\frac{dV_{S}}{ds}(s)>0$ and λ > 0 ensure existence of a solution *σ*(*t*, λ) of (35), and $\frac{d^{2}V_{S}}{ds^{2}}<0$ (which is equivalent to *α*_*S*_ > 0) ensures uniqueness and continuity. The two “barrier” requirements, (36) and (37), ensure positivity and boundedness of *σ*(*t*, λ). In order to derive (38), we differentiate the expression on the left-hand side of (35) with respect to *t* and obtain

$$\begin{array}{c} 0=\frac{d}{dt}(\frac{e^{-(n-r)\cdot t}}{\left(\gamma +r\right)}\cdot \frac{dV_{S}}{ds}(\sigma (t)))=\frac{e^{-(n-r)\cdot t}}{\left(\gamma +r\right)}\cdot \alpha_{S}(\sigma )\cdot \frac{\frac{dV_{S}}{ds}}{\sigma }\cdot (\frac{\left(r-n\right)}{\alpha_{S}(\sigma )}\cdot \sigma -\frac{d\sigma (t)}{dt}) \end{array}$$

Since $\frac{e^{-(n-r)\cdot t}}{\left(\gamma +r\right)}\cdot \alpha_{S}(\sigma )\cdot \frac{\frac{dV_{S}}{ds}}{\sigma }\neq 0$, we have

$$\begin{array}{c} \frac{d\sigma (t)}{dt}=\frac{\left(r-n\right)}{\alpha_{S}(\sigma )}\cdot \sigma , \end{array}$$ (39)

and now we transform (2) as follows:

$$\begin{array}{c} c(t)=\frac{1}{f}\cdot (\frac{d\sigma }{dt}+\gamma \cdot \sigma )=\frac{1}{f}\cdot (\frac{\left(r-n\right)}{\alpha_{S}(\sigma )}\cdot \sigma +\gamma \cdot \sigma )=\frac{r+\alpha_{S}\cdot \gamma -n}{f\cdot \alpha_{S}}\cdot \sigma (t) \end{array}$$

This completes the derivation of (38).

**Remark 18** *In* [4] *a less restrictive assumption is used, namely* $\frac{dV_{S}}{ds}(s_{M})=0$ *with* 0 < *s*_*M*_ < ∞ *instead of our* (37). *Our results could be extended, with some modifications, to that case too*.

**Definition 19** *The solution of* Eq (35), *σ* (*t*, λ), *whose existence and basic properties are as described in Lemma 17, is called the general solution*.

Now with the help of Lemma 17 we can derive the properties of subintervals of [0, *T*] in addition to those we derived in Proposition 16.

**Proposition 20** *Let* [*s*, λ_*c*_, λ_*W*_] ∈ *BV* ([0, *T*] → *R*) × *Lip* ([0, *T*] → *R*) × *R be a solution of Problem 10*. *In every subinterval of* [0, *T*] *in which* λ_*c*_ > 0, *the satiation is of the form s*(*t*) = *const* ⋅ *e*^−*γ*⋅*t*^ *and the following hold*:

1. *In every open subinterval of* [0, *T*] *in which* λ_*c*_ = 0, *the optimal satiation is s*(*t*) = *σ*(*t*, λ_*W*_) *if for all s*
  $$\begin{array}{c} n<r+\alpha_{S}\cdot \gamma . \end{array}$$ (40)
2. *Consumption c*(*t*) *is* 0 *everywhere in* (0, *T*) *if for all s*
  $$\begin{array}{c} n>r+\alpha_{S}\cdot \gamma . \end{array}$$ (41)

**Proof**. If λ_*c*_ > 0 in a (relatively) open subinterval, the first KKT condition in (17) implies that *c* = 0 in that subinterval, hence the solution of (2) is of the form *s*(*t*) = *const* ⋅ *e*^−*γ*⋅*t*^. If λ_*c*_ = 0 on a closed subinterval, Eq (20) simplifies to (35) with λ = λ_*W*_, hence *s*(*t*) = *σ*(*t*, λ_*W*_). If (40) holds for all *s*, the corresponding consumption is positive. However, if (41) holds for all *s*, then (38) implies that *c* < 0, which violates requirement (5), hence consumption must be everywhere 0 in (0, *T*).

Proposition 20 also shows that *s*(*t*) must be continuously differentiable everywhere, except possibly at the endpoints of the open subintervals in which λ_*c*_ = 0, where there may be discontinuities in satiation and gulps of consumption; however, as will be proved in Lemma 24, this possibility is excluded. Proposition 20 also shows that the solutions can be divided into two groups. Each of these two groups corresponds to a particular pair of sufficient conditions, one pair being the conditions given in inequalities (32) and (40), and the other pair being the conditions given in inequalities (33) and (41). Hence these two groups of solutions will be investigated separately. The structure of solutions of Problem 10 under (32) and (40) is as described in Theorem 25 (Section 6.1), and the structure of solutions of Problem 10 under (33) and (41) is as described in Theorem 21 (Section 6.2).

### 6.1 The structure and existence of solutions for large future discount

When the future discount rate *n* is large, intuition suggests that all the consumption should take place at the beginning of the interval [0, *T*]. This is confirmed by the following theorem:

**Theorem 21 (*Explicit solution when the future discount is large*)** *If both V and V*_*S*_ *are increasing, concave-down utilities and* (33) *and* (41) *hold for all s* ≥ 0, *then the optimal solution* [*s*, λ_*c*_, λ_*W*_] ∈ *BV* ([0, *T*] → *R*) × *Lip*([0, *T*] → *R*) × *R is as follows*: *s is given by*

$$\begin{array}{c} s(t)=\{\begin{array}{cc} s_{0}\;, & t=0\\ s_{+}\cdot e^{-\gamma \cdot t}, & t\in (0,T], \end{array} \end{array}$$ (42)

*where s*_+_ = *f* ⋅ *W* + *s*_0_ (see (44)); λ_*W*_ *is given by* (49), λ_*W*_ > 0; *and* λ_*c*_ *is given by* (53), λ_*c*_ > 0. *All the consumption takes place in a single gulp at t* = 0.

**Proof**. By (33), the second part of Proposition 14 implies that there is no gulp of consumption at *t* = *T*, and Lemma 20 states that there is no consumption in (0, *T*), hence all the consumption must happen in a gulp at *t* = 0. Therefore, satiation is of the form (42), and the corresponding consumption is given by

$$\begin{array}{c} c(t)=\delta_{0}(t)\cdot \frac{s_{+}-s_{0}}{f}=\{\begin{array}{cc} \frac{s_{+}-s_{0}}{f}, & t=0\\ 0\;, & t\in (0,T] \end{array} \end{array}$$ (43)

The constant *s*_+_ can be calculated using the wealth constraint (13):

$$\begin{array}{c} s_{+}=f\cdot W+s_{0}>s_{0} \end{array}$$ (44)

The condition *s*_+_ > *s*_0_ ensures that the gulp of consumption at *t* = 0 is positive. We need to show that λ_*W*_ > 0 and λ_*c*_ > 0 in (0, *T*). For λ_*W*_ and λ_*c*_, we have the following equations from Problem 10:

For *t* = 0, by the first KKT condition in (17) and the fact that there is a gulp of consumption (*c*(0) > 0):

$$\begin{array}{c} \lambda_{c}\left(0\right)=0 \end{array}$$ (45)

For *t* ∈ (0, *T*), from (20):

$$\begin{array}{c} 0=e^{-n\cdot t}\cdot \frac{dV_{S}}{ds}(s_{+}\cdot e^{-\gamma \cdot t})-\lambda_{W}\cdot e^{-r\cdot t}\cdot \left(\gamma +r\right)+(-\frac{d\lambda_{c}}{dt}+\lambda_{c}\cdot \gamma ) \end{array}$$ (46)

For *t* = *T*, from (19):

$$\begin{array}{c} 0=e^{-n\cdot T}\cdot \frac{dV}{ds}(s_{+}\cdot e^{-\gamma \cdot T})-\lambda_{W}\cdot e^{-r\cdot T}+\lambda_{c}\left(T\right) \end{array}$$ (47)

We need to prove that λ_*c*_(*t*) > 0 in (0,*T*] and λ_*W*_ > 0. The solution of (46), together with the initial condition (45), is

$$\begin{array}{c} \lambda_{c}(t)=e^{\gamma \cdot t}\cdot \int_{0}^{t}e^{-(n+\gamma )\cdot \tau }\cdot \frac{dV_{S}}{ds}(s_{+}\cdot e^{-\gamma \cdot \tau })\cdot d\tau -\lambda_{W}\cdot e^{\gamma \cdot t}\cdot (1-e^{-(r+\gamma )\cdot t}) \end{array}$$ (48)

After substitution of this into the boundary condition (47), we obtain an equation for λ_*W*_:

$$\begin{array}{c} \lambda_{W}=e^{-(n+\gamma )\cdot T}\cdot \frac{dV}{ds}(s_{+}\cdot e^{-\gamma \cdot T})+\int_{0}^{T}e^{-(n+\gamma )\cdot \tau }\cdot \frac{dV_{S}}{ds}(s_{+}\cdot e^{-\gamma \cdot \tau })\cdot d\tau \end{array}$$ (49)

This implies that λ_*W*_ > 0, since $\frac{dV}{ds}>0$ and $\frac{dV_{S}}{ds}>0$. This expression can be simplified. First, note that

$$\begin{array}{c} \frac{d}{d\tau }(e^{-(n+\gamma )\cdot \tau }\cdot \frac{dV}{ds}(s_{+}\cdot e^{-\gamma \cdot \tau }))=-e^{-(n+\gamma )\cdot \tau }\cdot \frac{dV_{S}}{ds}(s_{+}\cdot e^{-\gamma \cdot \tau }), \end{array}$$ (50)

hence the first term on the right-hand side of (49) can be transformed as follows:

$$\begin{array}{c} e^{-(n+\gamma )\cdot T}\cdot \frac{dV}{ds}(s_{+}\cdot e^{-\gamma \cdot T})=\frac{dV}{ds}(s_{+})-\int_{0}^{T}e^{-(n+\gamma )\cdot \tau }\cdot \frac{dV_{S}}{ds}(s(t))\cdot d\tau \end{array}$$ (51)

Therefore, by (10),

$$\begin{array}{ccc} \lambda_{W} & = & \frac{dV}{ds}(s_{+})-\int_{0}^{T}e^{-(n+\gamma )\cdot \tau }\cdot (n+\gamma \cdot (1-\alpha ))\cdot \frac{dV}{ds}\cdot d\tau \\ & & +\int_{0}^{T}e^{-(n+\gamma )\cdot \tau }\cdot (n+\gamma \cdot (1-\alpha ))\cdot \frac{dV}{ds}\cdot d\tau \\ & = & \frac{dV}{ds}(s_{+})>0 \end{array}$$

Now (48) takes the form

$$\begin{array}{c} \lambda_{c}(t)=e^{\gamma \cdot t}\cdot \int_{0}^{t}e^{-(n+\gamma )\tau }\cdot \frac{dV_{S}}{ds}(s_{+}\cdot e^{-\gamma \cdot \tau })\cdot d\tau -\frac{dV}{ds}(s_{+})\cdot e^{\gamma \cdot t}+\frac{dV}{ds}(s_{+})\cdot e^{-r\cdot t} \end{array}$$ (52)

Integration of (50) on [0, *t*] produces

$$\begin{array}{c} e^{-(n+\gamma )\cdot t}\cdot \frac{dV}{ds}(s(t))-\frac{dV}{ds}(s_{+})=-\int_{0}^{t}(e^{-(n+\gamma )\cdot \tau }\cdot \frac{dV_{S}}{ds}(s_{+}\cdot e^{-\gamma \cdot \tau }))\cdot d\tau , \end{array}$$

hence

$$\begin{array}{c} \frac{dV}{ds}(s_{+})=e^{-(n+\gamma )\cdot t}\cdot \frac{dV}{ds}(s(t))+\int_{0}^{t}e^{-(n+\gamma )\cdot \tau }\cdot \frac{dV_{S}}{ds}(s_{+}\cdot e^{-\gamma \cdot \tau })\cdot d\tau , \end{array}$$

and now (52) takes the form

$$\begin{array}{ccc} \lambda_{c}(t) & = & \frac{dV}{ds}(s_{+})\cdot e^{-r\cdot t}-e^{-nt}\cdot \frac{dV}{ds}(s_{+}\cdot e^{-\gamma t})\\ & = & -e^{-r\cdot t}\cdot [e^{-(n-r)t}\cdot \frac{dV}{ds}(s_{+}\cdot e^{-\gamma t})-\frac{dV}{ds}(s_{+})]. \end{array}$$

Using transformations similar to those used in the proof of (51), we can prove the following:

$$\begin{array}{c} e^{-(n-r)\cdot t}\cdot \frac{dV}{ds}(s_{+}\cdot e^{-\gamma t})-\frac{dV}{ds}(s_{+})=\int_{0}^{t}(n-r-\alpha \cdot \gamma )\cdot e^{-(n-r)\cdot \tau }\cdot \frac{dV}{ds}(s_{+}\cdot e^{-\gamma \cdot \tau })\cdot d\tau , \end{array}$$

hence

$$\begin{array}{c} \lambda_{c}(t)=e^{-r\cdot t}\cdot \int_{0}^{t}(n-r-\alpha \cdot \gamma )\cdot e^{-(n-r)\cdot \tau }\cdot \frac{dV_{S}}{ds}(s_{+}\cdot e^{-\gamma \cdot \tau })\cdot d\tau , \end{array}$$ (53)

which is positive, thanks to (33) and the fact that $\frac{dV_{S}}{ds}>0$.

**Remark 22** *Since the objective function in Problem 10 is concave down, the sufficient conditions are also necessary, and there is only one solution. This result establishes the existence and uniqueness of solutions of the equivalent of Problem 1 in the class of BV functions, independent of the utility*.

### 6.2 Small future discount rate and solutions with positive distributed consumption

In this section we discuss the structure of solutions under assumptions (32) and (40). The results of this section permit us to prove the existence of solutions in Section 7. We begin with a lemma, which we will use in the proof of Theorem 25, that permits us to reduce the number of intervals as described in Propositions 16 and 20 (with or without consumption) to no more than two.

**Lemma 23** *Let* [*s*, λ_*c*_, λ_*W*_] ∈ *BV* ([0, *T*] → *R*) × *Lip* ([0, *T*] → *R*) × *R be a solution of Problem 10, and let α*_*S*_(*s*) > 0. *Suppose there are two times t*_1_, *t*_2_ *with* 0 ≤ *t*_1_ < *t*_2_ ≤ *T at which* λ_*c*_(*t*_1_) = λ_*c*_(*t*_2_) = 0. *Then* λ_*c*_(*t*) = 0, *s*(*t*) = *σ*(*t*, λ_*W*_) (*the general solution from Definition 19*), *and c*(*t*) > 0 *for all t* ∈ (*t*_1_, *t*_2_), *hence the non-negative Borel measure c is continuous with respect to Lebesgue measure in* (*t*_1_, *t*_2_).

**Proof**. In (*t*_1_, *t*_2_), *s*(*t*) is a solution of Eq (20), while the general solution *σ*(*t*, λ_*W*_) is a solution of (35). We eliminate λ_*W*_ from these two equations by subtraction to obtain

$$\begin{array}{c} 0=e^{-n\cdot t}\cdot (\frac{dV_{S}}{ds}(s(t))-\frac{dV_{S}}{ds}(\sigma (t,\lambda_{W})))+(-\frac{d\lambda_{c}}{dt}+\lambda_{c}\cdot \gamma ). \end{array}$$

Multiplying this equation by (*s*(*t*) − *σ*(*t*, λ_*W*_)), and with the help of the Fundamental Theorem of Calculus, we obtain

$$\begin{array}{ccc} 0 & = & e^{-n\cdot t}\cdot {\left(s(t)-\sigma (t,\lambda_{W})\right)}^{2}\cdot \int_{0}^{1}\frac{d^{2}V_{S}}{ds^{2}}((\sigma (t,\lambda_{W}))+x\cdot (s(t))-(\sigma (t,\lambda_{W})))\cdot dx\\ & & \;-\frac{d}{dt}[(s(t)-\sigma (t,\lambda_{W}))\cdot \lambda_{c}]\\ & & \;+\frac{d}{dt}(s(t)-\sigma (t,\lambda_{W}))\cdot \lambda_{c}+(s(t)-\sigma (t,\lambda_{W}))\cdot \lambda_{c}\cdot \gamma \end{array}$$

Next, we integrate this over (*t*_1_, *t*_2_) to obtain

0=∫(t1,t2){[d2VSds2(σ(t,λW)+x·(s(t)−σ(t,λW)))][e−(n+γ)·t·(s(t)−σ(t,λW))2]·∫01[d2VSds2(σ(t,λW)+x·(s(t)−σ(t,λW)))]·dx}·dt+∫(t1,t2)[ddt(s(t)−σ(t,λW))+(s(t)−σ(t,λW))·γ]·λc·dt, (54)

since the boundary terms ${\left[(s(t)-\sigma (t,\lambda_{W}))\cdot \lambda_{c}(t)\right]}_{t=t_{1}}^{t=t_{2}}$ vanish, thanks to the fact that λ_*c*_(*t*_1_) = λ_*c*_(*t*_2_) = 0. Next, thanks to (2) and (39),

$$\begin{array}{c} \frac{d}{dt}(s(t)-\sigma (t,\lambda_{W}))=(c(t)-\gamma \cdot s(t)-\frac{\left(r-n\right)}{\alpha_{S}(\sigma (t,\lambda_{W}))}\cdot \sigma (t,\lambda_{W})), \end{array}$$

hence the second integral in (54) can be rewritten as follows:

$$\begin{array}{ccc} & & \int_{\left(t_{1},t_{2}\right)}(\frac{d}{dt}(s(t)-\sigma (t,\lambda_{W}))\cdot \lambda_{c}+\gamma \cdot (s(t)-\sigma (t,\lambda_{W}))\cdot \lambda_{c})\cdot dt\\ & = & \int_{\left(t_{1},t_{2}\right)}c(t)\cdot \lambda_{c}\cdot dt-\int_{\left(t_{1},t_{2}\right)}[\frac{\left(r-n\right)}{\alpha_{S}(\sigma (t,\lambda_{W}))}+\gamma ]\cdot \sigma (t,\lambda_{W})\cdot \lambda_{c}\cdot dt\\ & = & -\int_{\left(t_{1},t_{2}\right)}\frac{r+\gamma \cdot \alpha_{S}-n}{\alpha_{S}}\cdot \sigma (t,\lambda_{W})\cdot \lambda_{c}\cdot dt, \end{array}$$

since *c*(*t*) ⋅ λ_*c*_(*t*) = 0, thanks to the first KKT condition in (17). Hence we obtain

∫(t1,t2){[∫01d2VSds2(σ(t,λW)+x·(s(t)−σ(t,λW)))·dx][e−(n+γ)·t·(s(t)−σ(t,λW))2]·[∫01d2VSds2(σ(t,λW)+x·(s(t)−σ(t,λW)))·dx]}·dt=∫(t1,t2)(r+αS·γ−n)αS·σ(t,λW)·λc(t)·dt

The left-hand side is less than or equal to 0, thanks to the fact that $\frac{d^{2}V_{S}}{ds^{2}}<0$, while the right-hand side is greater than or equal to 0, thanks to (40). Moreover, *σ*(*t*, λ_*W*_) > 0 by Lemma 17, and λ_*c*_(*t*) ≥ 0. Hence both sides must be equal to 0. This is possible only if (*s*(*t*) − *σ*(*t*, λ_*W*_)) = 0, hence *c* > 0, thanks to (38) and (40), so λ_*c*_(*t*) = 0 in (*t*_1_, *t*_2_).

Lemma 23 implies that between any two open intervals with *c*(*t*) > 0 and λ_*c*_ = 0 there cannot be any interval with λ_*c*_ > 0, which in turn implies that there can be at most one interval with λ_*c*_ > 0 and at most one interval with λ_*c*_ = 0. This fact is used in the proof of Theorem 25. The next lemma, which is also used in that proof, permits us to reduce the number of gulps of consumption to no more than two.

**Lemma 24** *Let* [*s*, λ_*c*_, λ_*W*_] ∈ *BV*([0, *T*] → *R*) × *Lip*([0, *T*] → *R*) × *R be a solution of Problem 10*. *If ε* > 0 *and T*_*s*_ ∈ (0, *T*), *and T*_*s*_ *is a switching boundary between an interval* (*T*_*s*_ − *ε*, *T*_*s*_) *in which c* = 0 *and* λ_*c*_ > 0 *and an interval* [*T*_*s*_, *T*_*s*_ + *ε*) *in which c* > 0 *and* λ_*c*_ = 0, *then the satiation s*(*t*) *is continuous at the switching time T*_*s*_ *and there is no gulp of consumption at T*_*s*_. *The non-negative Borel measure c is continuous with respect to Lebesgue measure in* (0, *T*).

**Proof**. If there is a gulp of consumption $\frac{s(T_{s}^{+})-s(T_{s}^{-})}{f}\neq 0$ at *T*_*s*_, then inequality constraint (5) implies that it must be non-negative. Since *f* > 0, it must be the case that $s(T_{s}^{+})\geq s(T_{s}^{-})$. Therefore, in order to prove that $s(T_{s}^{+})=s(T_{s}^{-})$, we have to prove that $s(T_{s}^{+})\leq s(T_{s}^{-})$. In (*T*_*s*_ − *ε*, *T*_*s*_) satiation *s*(*t*) satisfies Eq (2) with *c* = 0, hence $s(t)=const\cdot e^{-\gamma \cdot (t-T_{s})}=s(T_{s}^{-})\cdot e^{-\gamma \cdot (t-T_{s})}$ in (*T*_*s*_ − *ε*, *T*_*s*_), where $s(T_{s}^{-})={\text{lim}}_{t\to T_{s},t<T_{s}}s(t)$. In [*T*_*s*_, *T*_*s*_ + *ε*) the satiation *s*(*t*) satisfies Eq (20) with λ_*c*_(*t*) = 0, that is, it satisfies Eq (35), hence $\lambda_{W}=\lim_{t\to T_{s},t>T_{s}}\frac{e^{-(n-r)\cdot t}}{e^{-(n-r)\cdot t}(\gamma +r)}\cdot \frac{dV_{S}}{ds}(s(t))=\frac{e^{-(n-r)\cdot T_{s}}}{\left(\gamma +r\right)}\cdot \frac{dV_{S}}{ds}(s(T_{s}^{+}))$, where $s(T_{s}^{+})=\lim_{t\to T_{s},t>T_{s}}s(t)$. Since λ_*c*_(*t*) ≥ 0 in (*T*_*s*_ − *ε*, *T*_*s*_) and λ_*c*_(*T*_*s*_) = 0, we have $0\geq \frac{d\lambda_{c}}{dt}(T_{s}^{-})$. In (*T*_*s*_ − *ε*, *T*_*s*_) the Lagrange multiplier λ_*c*_(*t*) satisfies Eq (20) with λ_*c*_(*t*) ≥ 0, thanks to the second KKT condition in (17), hence

$$\begin{array}{ccc} 0 & \geq & \frac{d\lambda_{c}}{dt}(T_{s}^{-})\\ & = & e^{-n\cdot T_{s}}\cdot \frac{dV_{S}}{ds}(s(T_{s}^{-}))-\lambda_{W}\cdot e^{-r\cdot T_{s}}\cdot (\gamma +r)+\lambda_{c}(T_{s})\cdot \gamma \\ & = & e^{-n\cdot T_{s}}\cdot [\frac{dV_{S}}{ds}(s(T_{s}^{-}))-\frac{dV_{S}}{ds}(s(T_{s}^{+}))] \end{array}$$

for λ_*c*_(*T*_*s*_) = 0, because λ_*c*_(*t*) is continuous and λ_*c*_(*t*) = 0 for *t* > *T*_*s*_; therefore, $\frac{dV_{S}}{ds}(s(T_{s}^{+}))\geq \frac{dV_{S}}{ds}(s(T_{s}^{-}))$. Since $\frac{d^{2}V_{S}}{ds^{2}}<0$, the above is possible only if $s(T_{s}^{+})\leq s(T_{s}^{-})$, which, together with the opposite inequality ($s(T_{s}^{+})-s(T_{s}^{-})\geq 0$), implied by *c* > 0 and *f* > 0, implies that $s(T_{s}^{+})=s(T_{s}^{-})$, and hence that *s*(*t*) is continuous at *t* = *T*_*s*_. The consumption *c*(*t*) is 0 in every relatively open interval where λ_*c*_(*t*) > 0, hence it is trivially continuous with respect to Lebesgue measure there, and similarly it is continuous with respect to Lebesgue measure in intervals where *c*(*t*) > 0 by Lemma 23. Now continuity of satiation *s* at the boundaries between these two types of intervals, which is as described in Proposition 16, implies continuity of consumption *c* with respect to Lebesgue measure throughout the interval (0, *T*).

Now we can prove the main result of this section.

**Theorem 25 (*The structure of solutions when the future discount is small*)** *Let* [*s*, λ_*c*_, λ_*W*_] ∈ *BV* ([0, *T*] → *R*) × *Lip*([0, *T*] → *R*) × *R be a solution of Problem 10*. *If* (32) *and* (40) *hold, then there is a switching time T*_*s*_ ∈ [0, *T*] *such that c*(*t*) = 0 *and* λ_*c*_(*t*) > 0 *for t* ∈ [0, *T*_*s*_); $c(t)=\frac{r+\alpha_{S}\cdot \gamma -n}{\alpha_{S}}\cdot s(t)>0$ *and* λ_*c*_(*t*) = 0 *for t* ∈ [*T*_*s*_, *T*]; *and s*(*t*) *is continuous at t* = *T*_*s*_ *if T*_*s*_ < *T*. *There is always a gulp of consumption at t* = *T*. *If T*_*s*_ > 0, *there is no gulp of consumption at t* = 0; *if T*_*s*_ = 0, *a gulp of consumption is possible*.

**Proof**. Suppose [*s*, λ_*c*_, λ_*W*_] ∈ *BV* ([0, *T*] → *R*) × *Lip*([0, *T*] → *R*) × *R* is a solution of Problem 10. By Proposition 14, there is a gulp of consumption at *t* = *T*, and hence the first KKT condition in (17) implies that λ_*c*_(*T*) = 0. By Proposition 16, there may be a countable set of closed intervals and isolated points where λ_*c*_ = 0, and any two of these are separated by an open interval where λ_*c*_ > 0. Let *T*_*s*_ = inf{*t* ∈ [0, *T*]|λ_*c*_ (*t*) = 0}. Since λ_*c*_ is continuous (Lemma 15), λ_*c*_ (*T*_*s*_) = 0. Now Proposition 23 implies that *c*(*t*) > 0 for all *t* ∈ [*T*_*s*_, *T*], hence the first KKT condition in (17) implies that λ_*c*_(*t*) = 0 in [*T*_*s*_, *T*]. Eq (38) implies that $c(t)=\frac{r+\alpha_{S}\cdot \gamma -n}{\alpha_{S}}\cdot s(t)$. The definition of *T*_*s*_ implies that λ_*c*_(*t*) > 0 and *c*(*t*) = 0 for all *t* ∈ [0, *T*_*s*_). If *T*_*s*_ < *T* then the continuity of *s*(*t*) at *t* = *T*_*s*_, and hence the absence of a gulp of consumption at *t* = *T*_*s*_, follows from Lemma 24. If there is a gulp of consumption at *t* = 0, then *T*_*s*_ is necessarily 0, hence there is no gulp of consumption at *t* = 0 if *T*_*s*_ > 0.

Theorem 25 establishes the existence of a unique solution of Problem 10. Since both of the utilities *V* and *V*_*S*_ are assumed to be concave down, every solution of boundary value Problem 10 is also a maximizer of the functional in (1) in Problem 1 on the BV space. Downward concavity also implies uniqueness; see [13]. In addition, Theorem 25 states that there must be a gulp of consumption at *t* = *T*; that if there is a gulp of consumption at *t* = 0, then *c*(*t*) > 0 in all of [0, *T*]; and that if there is no gulp of consumption at *t* = 0, there may be an initial interval [0, *T*_*s*_) without consumption(*c*(*t*) = 0), but *c*(*t*) > 0 in [*T*_*s*_, *T*], where *T*_*s*_ = *T* is not excluded. The structure of solutions is more complicated when the following two pairs of assumptions are relaxed: (33) and (41), and (32) and (40).

## 7 Existence of solutions for small future discount

With the help of the results of Section 6, we can reduce the problem of the existence of solutions to solving non-differential equations, and we prove their solvability here. We prove theorems that, with the help of the diagnostic profiles described in Section 7.1, allow us to use the problem data to determine the type of the solution.

Theorem 25 implies that under assumptions (32) and (40), the original problem (Problem 1) does have a unique solution and there are only three possible types of solutions of Problem 10, described as follows:

**Definition 26** *A solution is I-shaped (poor or over-satiated agent) when c* = 0 *and* λ_*c*_ > 0 *in* [0, *T*), *and all consumption is done in a single gulp at t* = *T*; *J-shaped (intermediate agent) when c* = 0 *and* λ_*c*_ > 0 *in* [0, *T*_*s*_), *and c* > 0 *and* λ_*c*_ = 0 *in* [*T*_*s*_, *T*], *and there is a gulp at t* = *T*; *U-shaped (rich or under-satiated agent) when there is a gulp at t* = 0, *c* > 0 *and* λ_*c*_ = 0 *in all of* [0, *T*], *and there is a gulp at t* = *T*.

Section 6 contains almost a complete proof of existence of a solution of Problem 10. The only missing part is the proof of the second KKT condition in (17): λ_*c*_ ≥ 0. We now reduce this question to a simple non-differential inequality.

**Lemma 27** *Suppose* (40) *holds for all s and that s*_0_ > 0. *Let* [*s*, λ_*c*_, λ_*W*_] ∈ *BV* ([0, *T*] → *R*) × *Lip*([0, *T*] → *R*) × *R be an I-shaped solution* (*T*_*s*_ = *T*) *or a J-shaped solution* (0 < *T*_*s*_ < *T*) *that satisfies all the requirements of Problem 10, except possibly the KKT condition* λ_*c*_ ≥ 0 (*see* (17)) *in* [0, *T*_*s*_) *with* 0 < *T*_*s*_ ≤ *T*. *Then* λ_*c*_(*t*) > 0 *in* [0, *T*_*s*_) *iff*

$$\begin{array}{c} \lambda_{W}>\frac{1}{\left(\gamma +r\right)}\cdot e^{-(n-r)\cdot T_{s}}\cdot \frac{dV_{S}}{ds}(s_{0}\cdot e^{-\gamma \cdot T_{s}}) \end{array}$$ (55)

**Proof**. Eq (20) for λ_*c*_ implies that in [0, *T*_*s*_)

$$\begin{array}{c} \frac{d\lambda_{c}(t)}{dt}-\lambda_{c}(t)\cdot \gamma =e^{-n\cdot t}\cdot \frac{dV_{S}}{ds}(s_{0}\cdot e^{-\gamma \cdot t})-\lambda_{W}\cdot e^{-r\cdot t}\cdot (\gamma +r) \end{array}$$ (56)

We need to prove that λ_*c*_ > 0 in [0, *T*_*s*_). Multiplying (56) by *e*^−*γ*⋅*t*^, we obtain

$$\begin{array}{c} \frac{d}{dt}(\lambda_{c}(t)\cdot e^{-\gamma \cdot t})=e^{-(\gamma +r)\cdot t}\cdot [e^{-(n-r)\cdot t}\cdot \frac{dV_{S}}{ds}(s_{0}\cdot e^{-\gamma \cdot t})-\lambda_{W}\cdot (\gamma +r)], \end{array}$$

hence it suffices to show that (λ_*c*_(*t*) ⋅ *e*^−*γ*⋅*t*^) > 0 in [0, *T*_*s*_). The derivative of the expression in square brackets can be written as

$$\begin{array}{c} e^{-(n-r)\cdot t}\cdot [r-n+\gamma \cdot \alpha_{S}]\cdot \frac{dV_{S}}{ds}(s_{0}\cdot e^{-\gamma \cdot t}), \end{array}$$

which is positive, thanks to $\frac{dV_{S}}{ds}(s)>0$ and (40), hence the expression in square brackets,

$$\begin{array}{c} e^{-(n-r)\cdot t}\cdot \frac{dV_{S}}{ds}(s_{0}\cdot e^{-\gamma \cdot t})-\lambda_{W}\cdot (\gamma +r), \end{array}$$

takes its largest value in [0, *T*_*s*_) at *t* = *T*_*s*_, and that value is

$$\begin{array}{c} e^{-(n-r)\cdot T_{s}}\cdot \frac{dV_{S}}{ds}(s_{0}\cdot e^{-\gamma \cdot T_{s}})-\lambda_{W}\cdot (\gamma +r). \end{array}$$

If this is positive, then $\frac{d}{dt}(\lambda_{c}(t)\cdot e^{-\gamma \cdot t})>0$ for all *t* close to but less than *T*_*s*_, hence $(\lambda_{c}(t)\cdot e^{-\gamma \cdot t})<(\lambda_{c}(T_{s})\cdot e^{-\gamma \cdot T_{s}})=0$ for all such *t*. Therefore, condition (55) is necessary for λ_*c*_(*t*) ≥ 0. It remains to show that this is also a sufficient condition. Suppose condition (55) holds. Since $\frac{d^{2}V_{S}}{ds^{2}}<0$, we obtain

$$\begin{array}{ccc} \frac{d}{dt}(\lambda_{c}(t)\cdot e^{-\gamma \cdot t}) & = & e^{-(\gamma +r)\cdot t}\cdot [e^{-(n-r)\cdot t}\cdot \frac{dV_{S}}{ds}(s_{0}\cdot e^{-\gamma \cdot t})-\lambda_{W}\cdot (\gamma +r)]\\ & \leq & e^{-(\gamma +r)\cdot t}\cdot [e^{-(n-r)\cdot T_{s}}\cdot \frac{dV_{S}}{ds}(s_{0}\cdot e^{-\gamma \cdot T_{s}})-\lambda_{W}\cdot (\gamma +r)]<0, \end{array}$$

hence $\frac{d}{dt}(\lambda_{c}(t)\cdot e^{-\gamma \cdot t})<0$ for *t* ∈ [0, *T*_*s*_). Therefore, (λ_*c*_(*t*) ⋅ *e*^−*γ*⋅*t*^) is a decreasing function, so $(\lambda_{c}(t)\cdot e^{-\gamma \cdot t})>(\lambda_{c}(T_{s})\cdot e^{-\gamma \cdot T_{s}})=0$ for all *t* ∈ [0, *T*_*s*_). Thus condition (55) is necessary and sufficient for λ_*c*_(*t*) > 0 in [0, *T*_*s*_).

**Remark 28** *If s*_0_ → 0, *then condition* (55) *cannot be satisfied, since that would imply that* $\lambda_{W}>\lim_{s_{0}\to 0}\frac{dV_{S}}{ds}(s_{0}\cdot e^{-\gamma \cdot T_{s}})=\lim_{s\to 0}\frac{dV_{S}}{ds}(s)=\infty$, *thanks to* (36). *Hence* λ_*W*_ *would not be real, so the interval* [0, *T*_*s*_) *must be empty, that is*, *T*_*s*_ = 0.

### 7.1 The diagnostic profiles

In order to describe how the data of the problem determine the structure of solutions, we introduce two diagnostic profiles; these are the solutions with wealth constraint (6) ignored.

**Definition 29** *If s*_0_ > 0, *then the lower diagnostic profile I/J* $\left[s^{l},\lambda_{c}^{l},\lambda_{W}^{l}\right]$ *(in between I-shaped solutions and J-shaped solutions; see Definition 26) for Problem 10 is*

$$\begin{array}{c} s^{l}(t)=\{\begin{array}{cc} s_{0}\cdot e^{-\gamma \cdot t}, & t\in [0,T)\\ s_{T}^{l}\;, & t=T, \end{array} \end{array}$$

*where* $s_{T}^{l}$ *is a solution of* (21) *for s*(*T*),

$$\begin{array}{c} e^{-(n-r)T}\cdot \frac{dV}{ds}(s_{T}^{l})=\lambda_{W}^{l}, \end{array}$$ (57)

*and where*

$$\begin{array}{c} \lambda_{W}^{l}=\frac{e^{-(n-r)T}}{\left(\gamma +r\right)}\cdot \frac{dV_{S}}{ds}(s_{0}\cdot e^{-\gamma \cdot T}) \end{array}$$ (58)

*and* $\lambda_{c}^{l}(t)$ *is the solution of the terminal-value problem*

$$\begin{array}{c} \lambda_{c}^{l}(T)=0,\;e^{-nt}\cdot \frac{dV_{S}}{ds}(s_{0}e^{-\gamma \cdot t})-\lambda_{W}^{l}\cdot e^{-r\cdot t}\cdot (\gamma +r)+(-\frac{d\lambda_{c}^{l}}{dt}+\lambda_{c}^{l}\cdot \gamma )=0. \end{array}$$

*The corresponding consumption is* $c^{l}(t)=\delta_{T}(t)\cdot \frac{\left(s_{T}^{l}-s_{0}\cdot e^{-\gamma \cdot T}\right)}{f}$, *and the wealth W*^*l*^ *necessary to sustain this level of consumption is*

$$\begin{array}{ccc} W^{l} & = & \int_{\left[0,T\right]}e^{-r\cdot t}\cdot c(t)\cdot dt=\int_{\left[0,T\right]}e^{-r\cdot t}\cdot \delta_{T}(t)\cdot \frac{\left(s_{T}^{l}-s_{0}\cdot e^{-\gamma \cdot T}\right)}{f}\cdot dt\\ & = & e^{-r\cdot T}\cdot \frac{\left(s_{T}^{l}-s_{0}\cdot e^{-\gamma \cdot T}\right)}{f} \end{array}$$ (59)

**Proposition 30** $\lambda_{c}^{l}>0$ *in* [0, *T*).

**Proof**. By Lemma 27, it suffices to prove that condition (55) holds, which follows from (58), since *T*_*s*_ = *T* in the context of that lemma.

**Definition 31** *If s*_0_ > 0, *then the upper diagnostic profile U/J* $\left[s^{u},\lambda_{c}^{u}=0,\lambda_{W}^{u}\right]$ *(in between U-shaped solutions and J-shaped solutions; see Definition 26) for Problem 10, without a gulp of consumption at t* = 0, *is*

$$\begin{array}{c} s^{u}(t)=\{\begin{array}{cc} \sigma (t,\lambda_{W}^{u}), & t\in [0,T)\\ s_{T}^{u}\;, & t=T \end{array}\},\lambda_{c}^{u}=0\;\text{in}\;[0,T], \end{array}$$

*where σ*(*t*, λ) *is as described in Definition 19 and* $\lambda_{W}^{u}$ is chosen so that $\sigma (0,\lambda_{W}^{u})=s_{0}$, *which by* (35) *is equivalent to* $\frac{1}{\left(\gamma +r\right)}\cdot \frac{dV_{S}}{ds}(s_{0})=\lambda_{W}^{u}$, *and* $s_{T}^{u}\;=s_{T}$, *where s*_*T*_ *is the solution of* Eq (19) *with* λ_*c*_ = 0: $\lambda_{W}^{u}=\frac{1}{\left(\gamma +r\right)}\cdot \frac{dV_{S}}{ds}(s_{0})=e^{-(n-r)T}\cdot \frac{dV}{ds}(s_{T}^{u})$. *The corresponding consumption is*

$$\begin{array}{c} c^{u}(t)=\frac{r+\alpha_{S}\cdot \gamma -n}{\alpha_{S}}\cdot \sigma (t,\lambda_{W}^{u})+\delta_{T}(t)\cdot \frac{\left(s_{T}^{u}-\sigma (T,\lambda_{W}^{u})\right)}{f}, \end{array}$$

*and the wealth W*^*u*^ *needed to sustain this level of consumption is*

$$\begin{array}{ccc} f\cdot W^{u} & = & \int_{\left[0,T\right]}e^{-r\cdot t}\cdot (\frac{ds^{u}}{dt}+\gamma \cdot s^{u})\cdot dt\\ & = & \int_{\left[0,T\right]}(\frac{d}{dt}(e^{-r\cdot t}\cdot s^{u})+e^{-r\cdot t}\cdot (\gamma +r)\cdot s^{u})\cdot dt\\ & = & e^{-r\cdot T}\cdot s_{T}^{u}-s_{0}+(\gamma +r)\cdot \int_{\left[0,T\right]}e^{-r\cdot t}\cdot \sigma (t,\lambda_{W}^{u})\cdot dt \end{array}$$ (60)

**Remark 32** *If s*_0_ = 0, *then it is reasonable to accept W*^*u*^ = *W*^*l*^ = 0, *s*_*u*_(*t*) = *s*_*l*_(*t*) = 0, *and* $\lambda_{W}^{u}=\lambda_{W}^{l}=+\infty$.

**Remark 33** *The discrete analogue of the upper diagnostic profile is the only solution exhibited in* [1]; *other solutions given there were obtained numerically*.

The rest of this section is devoted to proving the following: If *W* ≤ *W*^*l*^, the solution is I-shaped; if *W*^*l*^ < *W* ≤ *W*^*u*^, the solution is J-shaped; and if *W*^*u*^ < *W*, the solution is U-shaped. This is achieved by reduction of Problem 10 to a non-differential equation (Eq (68)) for J-shaped solutions, and to a non-differential equation (Eq (62)) for U-shaped solutions.

**Remark 34** *In Proposition 38 it is proved that W*^*u*^ > *W*^*l*^ *for s*_0_ > 0.

### 7.2 U-shaped solutions for a rich or under-satiated agent with wealth *W* ≥ *W*^*u*^

If *W* ≥ *W*^*u*^ and *s*_0_ ≥ 0, the solution [*s*, λ_*c*_, λ_*W*_] of Problem 10 can be constructed as follows:

$$\begin{array}{c} s(t)=\{\begin{array}{cc} s_{0}\;, & t=0\\ \sigma (t,\lambda_{W}), & t\in (0,T)\\ s_{T}(\lambda_{W}), & t=T, \end{array} \end{array}$$ (61)

where *σ*(0, λ) is as described in Definition 19, *s*(*T*) = *s*_*T*_(λ_*W*_), *s*_*T*_(λ_*W*_) is a solution of Eq (57), and λ_*W*_ is to be determined from the wealth constraint (13) reduced to the non-differential equation

$$\begin{array}{c} w^{r}(\lambda_{W})=W, \end{array}$$ (62)

where *w*^*r*^(λ_*W*_) is defined as follows:

$$\begin{array}{c} f\cdot w^{r}(\lambda_{W})=e^{-r\cdot T}\cdot s_{T}(\lambda_{W})-s_{0}+(\gamma +r)\cdot \int_{\left[0,T\right]}e^{-r\cdot t}\cdot \sigma (t,\lambda_{W})\cdot dt. \end{array}$$

Therefore, the existence of solutions in this case reduces to solving Eq (62), and solvability of this equation is established in the following proposition:

**Proposition 35** *If W* ≥ *W*^*u*^, *there is exactly one solution* λ_*W*_ *of* Eq (62). *In addition, the solution given by* (61) *is a solution of Problem 10 and also an optimal solution of Problem 1*. *If W* = *W*^*u*^, *this solution coincides with the upper diagnostic profile; see Definition 31*.

**Proof**. We need to prove the existence of a unique solution of Eq (62), and we need to prove that the consumption gulps, $\frac{\sigma (0,\lambda_{W})-s_{0}}{f}$ at *t* = 0 and $\frac{s_{T}-\sigma (T,\lambda_{W})}{f}$ at *t* = *T*, are positive. In order to prove uniqueness, we find the derivative $\frac{d}{d\lambda_{W}}(w^{r}(\lambda_{W}))$:

$$\begin{array}{c} f\cdot \frac{d}{d\lambda_{W}}(w^{r}(\lambda_{W}))=e^{-r\cdot T}\cdot \frac{d}{d\lambda_{W}}(s_{T}(\lambda_{W}))+(\gamma +r)\cdot \int_{\left[0,T\right]}e^{-r\cdot t}\cdot \frac{d}{d\lambda_{W}}(\sigma (t,\lambda_{W}))\cdot dt \end{array}$$

The derivative $\frac{d}{d\lambda_{W}}(s_{T}(\lambda_{W}))$ is calculated using Eq (57):

$$\begin{array}{c} \frac{d}{d\lambda_{W}}(s_{T}(\lambda_{W}))=\frac{1}{e^{-(n-r)\cdot T}\cdot \frac{d^{2}V}{ds^{2}}(s_{T})}<0. \end{array}$$ (63)

The derivative $\frac{d}{d\lambda_{W}}(\sigma (t,\lambda_{W}))$ is calculated using Eq (35):

$$\begin{array}{c} \frac{d}{d\lambda_{W}}(\sigma (t,\lambda_{W}))=\frac{e^{(n-r)\cdot t}\cdot (\gamma +r)}{\frac{d^{2}V_{S}}{ds^{2}}(\sigma (t,\lambda_{W}))}<0. \end{array}$$ (64)

Thus

$$\begin{array}{c} \frac{d}{d\lambda_{W}}(w^{r}(\lambda_{W}))<0 \end{array}$$ (65)

This implies existence and uniqueness of a solution of *w*(λ_*W*_) = *W* with $\lambda_{W}\leq \lambda_{W}^{u}$ whenever *W* ≥ *W*^*u*^, since ${\text{lim}}_{\lambda_{w}\to 0}\left(\sigma \left(t,\lambda_{w}\right)\right)=+\infty$ and ${\text{lim}}_{\lambda_{w}\to 0}\left(s_{T}\left(\lambda_{w}\right)\right)=+\infty$. Positivity of the gulp of consumption at *t* = *T* and the result that λ_*W*_ > 0 follow from Proposition 14. If *W* = *W*^*u*^, the solution coincides with the upper diagnostic profile from Definition 31, thanks to the uniqueness proved earlier, and there is no gulp of consumption at *t* = 0. Since $\lambda_{W}<\lambda_{W}^{u}$, it follows that if *W* > *W*^*u*^, then (64) implies that $s_{0}=\sigma (0,\lambda_{W}^{u})<\sigma (0,\lambda_{W})$, hence the gulp of consumption at *t* = 0 is positive.

The corresponding consumption is given by

$$\begin{array}{c} c(t)=\delta_{0}(t)\cdot \frac{\sigma (0,\lambda )-s_{0}}{f}+\frac{r+\alpha_{S}\cdot \gamma -n}{\alpha_{S}}\cdot \sigma (t,\lambda_{W})+\delta_{T}(t)\cdot \frac{s-\sigma (T,\lambda )}{f} \end{array}$$

The solution [*s*, λ_*c*_, λ_*W*_] of Problem 10 which is obtained in Proposition 35 is continuously differentiable in (0, *T*), consumption is positive in all of (0, *T*), there is a gulp of consumption at *t* = *T*, and there is a gulp of consumption at *t* = 0 if *W* > *W*^*u*^. If *W* = *W*^*u*^, this solution coincides with the upper diagnostic profile $\left[s^{u},\lambda_{c}^{u}=0,\lambda_{W}^{u}\right]$ given in Definition 31.

**Remark 36** *If s*_0_ = 0, *only this type of solution is possible and consumption is positive in all of* [0, *T*].

### 7.3 J-shaped solutions for an agent with intermediate wealth (*W*^*l*^ ≤ *W* < *W*^*u*^)

If *W*^*l*^ ≤ *W* ≤ *W*^*u*^ and *s*_0_ > 0, the solution [*s*, λ_*c*_, λ_*W*_] of Problem 10 can be constructed as follows:

$$\begin{array}{c} s(t)=\{\begin{array}{cc} s_{0}\cdot e^{-\gamma \cdot t}, & t\in [0,T_{s}]\\ \sigma (t,\lambda_{W}), & t\in (T_{s},T)\\ s_{T}\;, & t=T, \end{array} \end{array}$$ (66)

where *T*_*s*_ ∈ [0, *T*], *σ*(*t*, λ) is as described in Definition 19, and *s*(*T*) = *s*_*T*_ = *s*_*T*_ (λ_*W*_(*T*_*s*_)) is a solution of Eq (21), where λ_*W*_(*T*_*s*_) is given by (67) below, while λ_*c*_ has to be found using Eq (21) for *t* ∈ [0, *T*_*s*_] with the terminal condition λ_*c*_(*T*_*s*_) = 0. By Lemma 24, the satiation *s*(*t*) is differentiable in [0, *T*_*s*_) ∪ (*T*_*s*_, *T*) and continuous at *T*_*s*_. In addition, it has a discontinuity at *t* = *T* and no consumption in [0, *T*_*s*_). Continuity at *t* = *T*_*s*_ means that $\sigma (T_{s},\lambda_{W})=s_{0}\cdot e^{-\gamma \cdot T_{s}}$, which is equivalent to

$$\begin{array}{c} \lambda_{W}(T_{s})=\frac{e^{-(n-r)\cdot T_{s}}}{\left(\gamma +r\right)}\cdot \frac{dV_{S}}{ds}(s_{0}\cdot e^{-\gamma \cdot T_{s}}) \end{array}$$ (67)

by Lemma 17 and Definition 19. *T*_*s*_ must be determined using the wealth constraint (6), which, by a method similar to the one used to derive (13), can be transformed into

$$\begin{array}{c} W=w^{m}(T_{s}), \end{array}$$ (68)

where

$$\begin{array}{c} f\cdot w^{m}(T_{s})=e^{-r\cdot T}\cdot s_{T}(T_{s})-s_{0}\cdot e^{-(\gamma +r)\cdot T_{s}}+(\gamma +r)\cdot \int_{\left[T_{s},T\right]}e^{-r\cdot t}\cdot \sigma (t,\lambda_{W}(T_{s}))\cdot dt. \end{array}$$ (69)

Therefore, the existence of solutions of Problem 10 in this case reduces to solving a non-differential equation (Eq (68)). The rest of this subsection is devoted to the proof of existence of solutions of Eq (68). We first prove a lemma which will allow us to prove the uniqueness of the solution of (68) and the fact that *W*^*u*^ > *W*^*l*^.

**Lemma 37** $\frac{d}{dT_{s}}(w^{m}(T_{s}))<0$

**Proof**. The derivative $\frac{d}{dT_{s}}(w^{m}(T_{s}))$ can be calculated as follows:

f·ddTswm(Ts)=ddTs(λW(Ts))·[∫[Ts,T]e−r·t·ddλW(σ(t,λW))·dte−r·T·ddλW(sT(λW))+(γ+r)·∫[Ts,T]e−r·t·ddλW(σ(t,λW))·dt],

since $\sigma (T_{s},\lambda_{W}(T_{s}))=s_{0}\cdot e^{-\gamma \cdot T_{s}}$. The expression in square brackets is negative, because (63) and (64) are applicable. The derivative $\frac{d}{dT_{s}}(\lambda_{W}(T_{s}))$ can be calculated using (67):

$$\begin{array}{c} \frac{d}{dT_{s}}(\lambda_{W}(T_{s}))=\frac{e^{-(n-r)\cdot T_{s}}}{\left(\gamma +r\right)}\cdot \frac{dV_{S}}{ds}(s_{0}\cdot e^{-\gamma \cdot T_{s}})\cdot (r-n+\gamma \cdot \alpha_{s})>0, \end{array}$$

thanks to (40), hence $\frac{d}{dT_{s}}(w^{m}(T_{s}))<0$.

**Proposition 38** *W*^*u*^ > *W*^*l*^ *if s*_0_ > 0.

**Proof**. If *W* = *W*^*u*^ = *w*^*m*^(0), the solution constructed in Proposition 39 coincides with the upper diagnostic profile $\left[s^{u},\lambda_{c}^{u}=0,\lambda_{W}^{u}\right]$ from Definition 31. If *W* = *W*^*l*^ = *w*^*m*^(*T*), it coincides with the lower diagnostic profile $\left[s^{l},\lambda_{c}^{l},\lambda_{W}^{l}\right]$ from Definition 29. However, $\frac{d}{dT_{s}}(w^{m}(T_{s}))<0$ for all *T*_*s*_ ∈ (0, *T*) by Lemma 37, hence *W*^*u*^ > *W*^*l*^.

**Proposition 39** *If s*_0_ > 0 *and W*^*l*^ ≤ *W* ≤ *W*^*u*^, *there is exactly one solution* λ_*W*_ *of* Eq (68). *Thus the solution given in* (61) *is a solution of Problem 10 and also a maximizer of the functional in Problem 1*.

**Proof**. Existence follows from *w*^*m*^(*T*) = *W*^*l*^ ≤ *W* ≤ *W*^*u*^ = *w*^*m*^(0) and the Intermediate Value Theorem, and uniqueness follows from $\frac{d}{dT_{s}}(w^{m}(T_{s}))<0$. The fact that λ_*c*_(*t*) > 0 in [0, *T*_*s*_) has already been proved in Lemma 27.

The corresponding consumption is

$$\begin{array}{c} c(t)=\{\begin{array}{cc} 0, & t\in [0,T_{s})\\ \frac{r+\alpha_{S}\cdot \gamma -n}{\alpha_{S}}\cdot \sigma (t,\lambda_{W}), & t\in (T_{s},T) \end{array}\}+\delta_{T}(t)\cdot \frac{\left(s_{T}-\sigma (T,\lambda_{W})\right)}{f} \end{array}$$

### 7.4 I-shaped solutions for a poor or over-satiated agent with wealth *W* < *W*^*l*^

If *W* < *W*^*l*^ and *s*_0_ > 0, an explicit solution [*s*, λ_*c*_, λ_*W*_] can be constructed as follows:

$$\begin{array}{c} s(t)=\{\begin{array}{cc} s_{0}\cdot e^{-\gamma \cdot t}, & t\in [0,T)\\ s_{T}=f\cdot W\cdot e^{r\cdot T}+s_{0}\cdot e^{-\gamma \cdot T}, & t=T, \end{array} \end{array}$$

where *s*_*T*_ can be determined using the wealth constraint (13):

$$\begin{array}{ccc} f\cdot W & = & e^{-r\cdot T}\cdot s_{T}-s_{0}\cdot e^{-(\gamma +r)\cdot T}=e^{-r\cdot T}\cdot [s_{T}-s_{0}\cdot e^{-\gamma \cdot T}]\\ s_{T} & = & f\cdot W\cdot e^{r\cdot T}+s_{0}\cdot e^{-\gamma \cdot T}>s_{0}\cdot e^{-\gamma \cdot T}, \end{array}$$ (70)

and λ_*W*_ can be found using (21):

$$\begin{array}{c} \lambda_{W}=e^{-(n-r)T}\cdot \frac{dV}{ds}(s_{T})=e^{-(n-r)T}\cdot \frac{dV}{ds}(f\cdot W\cdot e^{r\cdot T}+s_{0}\cdot e^{-\gamma \cdot T}), \end{array}$$

while λ_*c*_ has to be found using Eq (21) and the terminal condition λ_*c*_(*T*) = 0.

**Proposition 40** λ_*c*_ > 0 *in* [0, *T*).

**Proof**. By Lemma 27, it suffices to prove that condition (55) holds, but this is equivalent to *W* < *W*^*l*^. Indeed, thanks to (59) and (70), the inequality *W* < *W*^*l*^ is equivalent to $\;s_{T}<s_{T}^{l}$. Since $\frac{d^{2}V}{ds^{2}}<0$, this is in turn equivalent to $\frac{dV}{ds}(s_{T})>\frac{dV}{ds}(s_{T}^{l})$, which implies that

$$\begin{array}{c} \lambda_{W}=e^{-(n-r)\cdot T}\cdot \frac{dV}{ds}(s_{T})>e^{-(n-r)\cdot T}\cdot \frac{dV}{ds}(s_{T}^{l})=\frac{e^{-(n-r)\cdot T}}{\left(\gamma +r\right)}\cdot \frac{dV_{S}}{ds}(s_{0}\cdot e^{-\gamma \cdot T}), \end{array}$$

thanks to (57) and (58). This proves that condition (55) holds, and hence that $\lambda_{c}^{l}>0$ in [0, *T*) by Lemma 27.

The corresponding consumption is $c(t)=\delta_{T}(t)\cdot \frac{\left(s_{T}-s_{0}\cdot e^{-\gamma \cdot T}\right)}{f}$. There is no consumption in [0, *T*), but satiation has a discontinuity at *t* = *T*, and all the consumption takes place in a gulp which is caused by this discontinuity if *W* > 0. If *W* = *W*^*l*^, this solution coincides with the lower diagnostic profile $\left[s^{l},\lambda_{c}^{l}=0,\lambda_{W}^{l}\right]$ given in Definition 29.

## 8 Construction of explicit optimal solutions

In the previous section we described how to reduce the problem of finding optimal solutions of Problem 1 to solving non-differential equations. With the help of this theory, in this section we construct explicit solutions for logarithmic and CRRA utilities.

### 8.1 Logarithmic utility

In this section we solve the appropriate non-differential equations in the case of logarithmic utility: *V*(*s*) = ln (*s*), $\frac{dV(s)}{ds}=s^{-1}$, *α* = 1. By (9), the derived utility is

$$\begin{array}{c} V_{S}(s)=V(s)\cdot n+\gamma \cdot s\cdot \frac{dV}{ds}(s)=\ln(s)\cdot n+\gamma \cdot s\cdot s^{-1}=\ln(s)\cdot n+\gamma , \end{array}$$

$\frac{dV_{S}}{ds}(s)=\frac{n}{s}$, *α*_*S*_ = 1. We first establish several useful results. Conditions (32) and (40) coincide and reduce to *n* < *r* + *γ*. Here we discuss only the solutions that satisfy this condition.

#### 8.1.1 The diagnostic profiles

Eq (35) for the diagnostic profile of satiation (see definition 29) and the boundary condition (21) for *s*_*T*_ take the following form:

$$\begin{array}{c} 0=e^{-n\cdot T}\cdot \frac{dV}{ds}(s_{T})-\lambda_{W}\cdot e^{-r\cdot T}=e^{-n\cdot T}\cdot [{\left(s_{T}\right)}^{-1}-\lambda_{W}\cdot e^{(n-r)\cdot T}]. \end{array}$$

Their solutions are

$$\begin{array}{c} \sigma (t,\lambda_{W})=\frac{1}{\lambda_{W}}\cdot \frac{n}{\gamma +r}\cdot e^{-(n-r)\cdot t},\;s_{T}=\frac{1}{\lambda_{W}}\cdot e^{-(n-r)\cdot T} \end{array}$$ (71)

From the initial condition $s_{0}=\sigma (0,\lambda_{W})={\left(\lambda_{W}^{u}\right)}^{-1}\cdot \frac{n}{\gamma +r}$, we obtain $\lambda_{W}^{u}=\frac{n}{(\gamma +r)\cdot s_{0}}$, hence

$$\begin{array}{c} s_{T}^{u}=\frac{1}{\lambda_{W}^{u}}\cdot e^{-(n-r)\cdot T}=s_{0}\cdot \frac{\gamma +r}{n}\cdot e^{-(n-r)\cdot T}. \end{array}$$

Note that $\lambda_{c}^{u}=0$, since consumption *c*(*t*) > 0 in all of [0, *T*], hence the upper diagnostic profile *s*^*u*^(*t*) from Definition 31 is

$$\begin{array}{c} s^{u}(t)=\{\begin{array}{cc} \sigma (t,\lambda_{W}^{u})=s_{0}\cdot e^{-(n-r)\cdot t}, & t\in [0,T)\\ s_{T}^{u}=s_{0}\cdot \frac{\gamma +r}{n}\cdot e^{-(n-r)\cdot T}, & t=T \end{array}\},\;\lambda_{c}^{u}=0\;\text{in}\;[0,T] \end{array}$$

The corresponding consumption is

$$\begin{array}{ccc} c^{u}(t) & = & \frac{r+\alpha_{S}\cdot \gamma -n}{\alpha_{S}}\cdot \sigma (t,\lambda_{W}^{u})+\delta_{T}(t)\cdot \frac{\left(s_{T}^{u}-\sigma (T,\lambda_{W}^{u})\right)}{f}\\ & = & s_{0}\cdot (r+\gamma -n)\cdot e^{-(n-r)\cdot t}+\delta_{T}(t)\cdot s_{0}\cdot \frac{\gamma +r-n}{n\cdot f}\cdot e^{-(n-r)\cdot T} \end{array}$$

By (60), the wealth necessary for *s*^*u*^ is

$$\begin{array}{ccc} f\cdot W^{u} & = & e^{-r\cdot T}\cdot s_{T}^{u}-s_{0}+\int_{\left[0,T\right]}e^{-r\cdot t}\cdot (\gamma +r)\cdot \sigma (t,\lambda_{W}^{u})\cdot dt\\ & = & s_{0}\cdot (\frac{\gamma +r}{n}-1)\\ & = & s_{0}\cdot \frac{\gamma +r-n}{n} \end{array}$$

By Eqs (58) and (57), *s*_*T*_ is a solution of $e^{-(n-r)T}\cdot \frac{dV}{ds}(s_{T}^{l})=\lambda_{W}^{l}=\frac{e^{-(n-r)T}}{\left(\gamma +r\right)}\cdot \frac{dV_{S}}{ds}(s_{0}\cdot e^{-\gamma \cdot T})$, hence $s_{T}^{l}=s_{0}\cdot e^{-\gamma \cdot T}\cdot \frac{\left(\gamma +r\right)}{n}$. Then the lower diagnostic profile from Definition 29 is

$$\begin{array}{c} s^{l}(t)=\{\begin{array}{cc} s_{0}\cdot e^{-\gamma \cdot t}, & t\in [0,T)\\ s_{T}=s_{0}\cdot e^{-\gamma \cdot T}\cdot \frac{\left(\gamma +r\right)}{n}, & t=T \end{array} \end{array}$$

The corresponding consumption is $c^{l}(t)=\delta_{T}(t)\cdot \frac{\left(s_{T}^{l}-s_{0}\cdot e^{-\gamma \cdot T}\right)}{f}=\delta_{T}(t)\cdot s_{0}\cdot e^{-\gamma \cdot T}\cdot \frac{\gamma +r-n}{n\cdot f}$. By (59), the wealth necessary for *s*^*l*^ is

$$\begin{array}{c} f\cdot W^{l}=e^{-r\cdot T}\cdot (s_{T}-s_{0}\cdot e^{-\gamma \cdot T})=s_{0}\cdot \frac{\gamma +r-n}{n}\cdot e^{-(\gamma +r)\cdot T}=f\cdot W^{u}\cdot e^{-(\gamma +r)\cdot T}<f\cdot W^{u} \end{array}$$

Here we will analyze only two cases: that of a normally satiated agent and that of a rich or over-satiated agent. These cases were discussed for general utilities in Section 7.4.

#### 8.1.2 J-shaped solutions for an agent with intermediate wealth (*W*^*l*^ ≤ *W* < *W*^*u*^)

If *W*^*l*^ ≤ *W* ≤ *W*^*u*^ and *s*_0_ > 0, the solution [*s*, λ_*c*_, λ_*W*_] is

$$\begin{array}{c} s(t)=\{\begin{array}{cc} s_{0}\cdot e^{-\gamma \cdot t}, & t\in [0,T_{s}]\\ \frac{1}{\lambda_{W}}\cdot e^{-(n-r)\cdot t}\cdot \frac{n}{\gamma +r}=s_{0}\cdot e^{-\gamma \cdot T_{s}}\cdot e^{-(n-r)\cdot (t-T_{s})}, & t\in (T_{s},T)\\ \frac{1}{\lambda_{W}}\cdot e^{-(n-r)\cdot T}=s_{0}\cdot e^{-\gamma \cdot T_{s}}\cdot e^{-(n-r)\cdot (T-T_{s})}\cdot \frac{\gamma +r}{n}, & t=T, \end{array} \end{array}$$

where $\frac{1}{\lambda_{W}}=s_{0}\cdot e^{-\gamma \cdot T_{s}}\cdot e^{(n-r)\cdot T}\cdot \frac{\gamma +r}{n}$ and *T*_*s*_ is to be determined from the wealth constraint (68), hence $f\cdot W=s_{0}\cdot e^{-(\gamma +r)\cdot T_{s}}\cdot \frac{\gamma +r-n}{n}$. We deem the above to be an equation for *T*_*s*_, hence the solution is $T_{s}=\frac{\text{ln}(\frac{W^{u}}{W})}{\left(\gamma +r\right)}=\frac{\text{ln}(\frac{s_{0}}{W}\cdot \frac{\gamma +r-n}{f\cdot n})}{\left(\gamma +r\right)}$. The corresponding consumption is

$$\begin{array}{ccc} c(t) & = & \left\{\begin{array}{cc} 0, & t\in [0,T_{s})\\ s_{0}\cdot \frac{r+\gamma -n}{n}\cdot e^{-\gamma \cdot T_{s}}\cdot e^{-(n-r)\cdot (t-T_{s})}, & t\in (T_{s},T) \end{array}\right\}\\ & & \;+\delta_{T}(t)\cdot s_{0}\cdot \frac{r+\gamma -n}{f\cdot n}\cdot e^{-\gamma \cdot T_{s}}\cdot e^{-(n-r)\cdot (T-T_{s})} \end{array}$$

#### 8.1.3 U-shaped solutions for a rich or under-satiated agent with wealth *W* ≥ *W*^*u*^

If *W* ≥ *W*^*u*^ and *s*_0_ ≥ 0, the solution [*s*, λ_*c*_, λ_*W*_] is

$$\begin{array}{c} s(t)=\{\begin{array}{cc} s_{0}\;, & t=0\\ s_{T}\cdot \frac{n}{\gamma +r}\cdot e^{\left(n-r)\cdot (T-t\right)}, & t\in (0,T)\\ s_{T}\;, & t=T \end{array}\},\;\lambda_{c}=0\;\text{in}\;[0,T], \end{array}$$

where *s*_*T*_ is to be determined from the wealth constraint (62) *f* ⋅ *W* + *s*_0_ = *s*_*T*_ ⋅ *e*^(*n*−*r*)⋅*T*^, hence

$$\begin{array}{c} s(t)=\{\begin{array}{cc} s_{0}\;, & t=0\\ (f\cdot W+s_{0})\cdot \frac{n}{\gamma +r}\cdot e^{-(n-r)\cdot t}, & t\in (0,T)\\ (f\cdot W+s_{0})\cdot e^{-(n-r)\cdot T}, & t=T \end{array} \end{array}$$

The corresponding consumption is

$$\begin{array}{ccc} c(t) & = & \delta_{0}(t)\cdot \frac{(f\cdot W+s_{0})\cdot \frac{n}{\gamma +r}-s_{0}}{f}\\ & + & (r+\gamma -n)\cdot (f\cdot W+s_{0})\cdot e^{-(n-r)\cdot t}\cdot \frac{n}{\gamma +r}\\ & + & \delta_{T}(t)\cdot (f\cdot W+s_{0})\cdot e^{-(n-r)\cdot T}\cdot \frac{\gamma +r-n}{f\cdot (\gamma +r)} \end{array}$$

### 8.2 Explicit optimal solutions for CRRA utility

In Section 7 we described how to reduce the problem of finding optimal solutions of Problem 1 to solving non-differential equations. In this section, we solve these equations for the case of CRRA utility $V(s)=\frac{s^{1-\alpha }-1}{1-\alpha }$, $\frac{dV(s)}{ds}=s^{-\alpha }$, where *α* is a constant, *α* ≠ 1. In this case we can use Eq (9) to find the derived utility *V*_*S*_:

$$\begin{array}{ccc} V_{S} & = & \frac{n+\gamma \cdot (1-\alpha )}{\left(1-\alpha \right)}\cdot s^{1-\alpha }-\frac{n}{1-\alpha },\;\\ \frac{dV_{S}}{ds} & = & (n+\gamma \cdot (1-\alpha ))\cdot \frac{dV}{ds}\\ & = & (n+\gamma \cdot (1-\alpha ))\cdot s^{-\alpha }. \end{array}$$

We first establish several useful results. Conditions (32) and (40) coincide and reduce to *n* < *r* + *γ* ⋅ *α*. The requirements on *V*_*S*_ and *α*_*S*_ in Remark 4 are satisfied. In addition, we need to assume that *n* − *r* ⋅ (1 − *α*) > 0. Eq (35) for the diagnostic profile of satiation and the boundary condition (21) on *s*_*T*_ take the following form:

$$\begin{array}{ccc} \frac{dV_{S}}{ds}(\sigma (t)) & = & \left(n+\gamma \cdot (1-\alpha ))\cdot \frac{dV}{ds}(\sigma (t)\right)\\ & = & (n+\gamma \cdot (1-\alpha ))\cdot {\left(\sigma (t)\right)}^{-\alpha }\\ & = & \lambda_{W}\cdot (\gamma +r)\cdot e^{(n-r)\cdot t},\\ 0 & = & e^{-n\cdot T}\cdot \frac{dV}{ds}(s_{T})-\lambda_{W}\cdot e^{-r\cdot T}\\ & = & e^{-n\cdot T}\cdot [{\left(s_{T}\right)}^{-\alpha }-\lambda_{W}\cdot e^{(n-r)\cdot T}] \end{array}$$

Their solutions are $\sigma (t,\lambda_{W})={\left(\lambda_{W}^{-1}\cdot Z\right)}^{\frac{1}{\alpha }}\cdot e^{-\frac{n-r}{\alpha }\cdot t}$, $s_{T}=\lambda_{W}^{-\frac{1}{\alpha }}\cdot e^{-\frac{n-r}{\alpha }\cdot T}=\sigma (T,\lambda_{W})\cdot Z^{-\frac{1}{\alpha }}$, where $Z=\frac{\left(n+\gamma \cdot (1-\alpha )\right)}{\left(\gamma +r\right)}$, hence we can also write

$$\begin{array}{c} \sigma (t,s_{T})=s_{T}\cdot Z^{\frac{1}{\alpha }}\cdot e^{\frac{n-r}{\alpha }\cdot (T-t)} \end{array}$$ (72)

#### 8.2.1 The diagnostic profiles

The upper diagnostic profile *s*^*u*^(*t*) from Definition 31 is

$$\begin{array}{c} s^{u}(t)=\{\begin{array}{cc} \sigma (t,\lambda_{W})=s_{0}\cdot e^{-\frac{n-r}{\alpha }\cdot t}, & t\in [0,T)\\ s_{T}^{u}=s_{0}\cdot Z^{-\frac{1}{\alpha }}\cdot e^{-\frac{n-r}{\alpha }\cdot T}, & t=T \end{array}\},\;\lambda_{c}^{u}=0\;\text{in}\;[0,T] \end{array}$$

The corresponding consumption is $c^{u}(t)=\frac{r+\alpha \cdot \gamma -n}{\alpha }\cdot s_{0}\cdot e^{-\frac{n-r}{\alpha }\cdot t}+\delta_{T}(t)\cdot \frac{s_{0}}{f}\cdot e^{-\frac{\left(n-r\right)}{\alpha }\cdot T}\cdot (Z^{-\frac{1}{\alpha }}-1)$. By (60), the wealth necessary for *s*^*u*^ is

$$\begin{array}{c} f\cdot W^{u}=s_{0}\cdot [\frac{\gamma \cdot \alpha +r-n}{n+r\cdot \alpha -r}+(Z^{-\frac{1}{\alpha }}-\alpha \cdot \frac{\gamma +r}{n-r\cdot (1-\alpha )})\cdot e^{-(r+\frac{n-r}{\alpha })\cdot T}] \end{array}$$

The lower diagnostic profile from Definition 29 is

$$\begin{array}{c} s^{l}(t)=\{\begin{array}{cc} s_{0}\cdot e^{-\gamma \cdot t}, & t\in [0,T)\\ s_{T}^{l}=s_{0}\cdot e^{-\gamma \cdot T}\cdot Z^{-\frac{1}{\alpha }}, & t=T \end{array}\},\;\lambda_{W}^{l}=s_{0}^{-\alpha }\cdot e^{(\gamma \cdot \alpha +r-n)\cdot T}\cdot Z^{-1} \end{array}$$

The corresponding consumption is $c^{l}(t)=\delta_{T}(t)\cdot s_{0}\cdot \frac{e^{-\gamma \cdot T}}{f}\cdot (\frac{\gamma \cdot \alpha +r-n}{\left(n+\gamma \cdot (1-\alpha )\right)}+Z^{-\frac{1}{\alpha }}-Z^{-1})$. By (59), the wealth necessary for *s*^*l*^ is

$$\begin{array}{c} f\cdot W^{l}=e^{-r\cdot T}\cdot (s_{0}\cdot e^{-\gamma \cdot T}\cdot Z^{-\frac{1}{\alpha }}-s_{0}\cdot e^{-\gamma \cdot T})=f\cdot W^{u}\cdot e^{-(\gamma +r)\cdot T}<f\cdot W^{u} \end{array}$$

Below, we discuss the cases of a normally satiated agent and a rich or under-satiated agent. The case of a poor or over-satiated agent was solved for general utilities in Section 7.4.

#### 8.2.2 J-shaped solutions for an agent with intermediate wealth (*W*^*l*^ ≤ *W* < *W*^*u*^)

If *W*^*l*^ ≤ *W* ≤ *W*^*u*^ and *s*_0_ > 0, the solution [*s*, λ_*c*_, λ_*W*_] is

$$\begin{array}{c} s(t)=\{\begin{array}{cc} s_{0}\cdot e^{-\gamma \cdot t}, & t\in [0,T_{s}]\\ s_{0}\cdot e^{-\gamma \cdot T_{s}}\cdot e^{-\frac{\left(n-r\right)}{\alpha }\cdot (t-T_{s})}, & t\in (T_{s},T)\\ s_{0}\cdot e^{-\gamma \cdot T_{s}}\cdot e^{-\frac{\left(n-r\right)}{\alpha }\cdot (T-T_{s})}\cdot Z^{-\frac{1}{\alpha }}, & t=T, \end{array} \end{array}$$

where *T*_*s*_ is to be determined using the wealth constraint (68), which can be transformed into

$$\begin{array}{c} f\cdot W=s_{0}\cdot e^{-(\gamma +r)\cdot T_{s}}\cdot [\frac{r+\gamma \cdot \alpha -n}{\left(n-r\cdot (1-\alpha )\right)}+e^{-(r+\frac{n-r}{\alpha })\cdot (T-T_{s})}\cdot E_{2}], \end{array}$$

and $E_{2}=({\left(\frac{\gamma +r}{n+\gamma \cdot (1-\alpha )}\right)}^{\frac{1}{\alpha }}-\alpha \cdot \frac{\gamma +r}{n-r\cdot (1-\alpha )})$. Note that *E*_2_ = 0 when *α* = 1. Thus we can use (13) to obtain an equation for *T*_*s*_:

$$\begin{array}{c} f\cdot W=s_{0}\cdot e^{-(\gamma +r)\cdot T_{s}}\cdot [\frac{r+\gamma \cdot \alpha -n}{n-r\cdot (1-\alpha )}+e^{-(r+\frac{n-r}{\alpha })\cdot (T-T_{s})}\cdot E_{2}] \end{array}$$

The corresponding consumption is

$$\begin{array}{ccc} c(t) & = & s_{0}\cdot e^{-\gamma \cdot T_{s}}\cdot [\{\begin{array}{cc} 0, & t\in [0,T_{s})\\ \frac{r+\alpha \cdot \gamma -n}{\alpha }\cdot e^{-\frac{\left(n-r\right)}{\alpha }\cdot (t-T_{s})}, & t\in (T_{s},T) \end{array}\}\\ & & \;\;\;+\delta_{T}(t)\cdot e^{-\frac{\left(n-r\right)}{\alpha }\cdot (T-T_{s})}\cdot \frac{1}{f}\cdot (Z^{-\frac{1}{\alpha }}-1)] \end{array}$$

#### 8.2.3 U-shaped solutions for a rich or under-satiated agent with wealth *W* ≥ *W*^*u*^

If *W* ≥ *W*^*u*^ and *s*_0_ ≥ 0, it is better to use (72) and write

$$\begin{array}{c} s(t)=\{\begin{array}{cc} s_{0}\;, & t=0\\ s_{T}\cdot Z^{\frac{1}{\alpha }}\cdot e^{+\frac{n-r}{\alpha }\cdot (T-t)}, & t\in (0,T)\\ s_{T}\;, & t=T \end{array}\},\;\;\lambda_{c}=0\;\text{in}\;[0,T], \end{array}$$

where $s_{T}=(f\cdot W+s_{0})\cdot B^{-1}\cdot e^{-\frac{n-r}{\alpha }\cdot T}$ is determined using the wealth constraint (62), $B=1-(1-e^{-(r+\frac{\delta -r}{\alpha })\cdot T})\cdot E_{3}$ (note that *B* = 1 when *α* = 1), and $E_{3}=1-Z^{\frac{1}{\alpha }}\cdot (\gamma +r)\cdot (r+\frac{n-r}{\alpha })=Z^{\frac{1}{\alpha }}\cdot E_{2}$, hence

$$\begin{array}{c} s(t)=\{\begin{array}{cc} s_{0}\;, & t=0\\ (f\cdot W+s_{0})\cdot B^{-1}\cdot Z^{\frac{1}{\alpha }}\cdot e^{-\frac{n-r}{\alpha }\cdot t}, & t\in (0,T)\\ (f\cdot W+s_{0})\cdot B^{-1}\cdot e^{-\frac{n-r}{\alpha }\cdot T}, & t=T \end{array} \end{array}$$

The corresponding consumption is

$$\begin{array}{ccc} c(t) & = & \delta_{0}(t)\cdot \frac{1}{f}\cdot ((f\cdot W+s_{0})\cdot B^{-1}\cdot Z^{\frac{1}{\alpha }}-s_{0})\\ & + & \frac{r+\alpha \cdot \gamma -n}{\alpha }\cdot (f\cdot W+s_{0})\cdot B^{-1}\cdot Z^{\frac{1}{\alpha }}\cdot e^{-\frac{n-r}{\alpha }\cdot t}\\ & + & \delta_{T}(t)\cdot \frac{1}{f}\cdot (f\cdot W+s_{0})\cdot B^{-1}\cdot e^{-\frac{n-r}{\alpha }\cdot T}\cdot (1-Z^{\frac{1}{\alpha }}) \end{array}$$

## 9 Conclusions and closing remarks

We investigated the structure and existence of solutions of the problem of consumption with satiation in continuous time for general utilities. We proved that the satiation function *s*(*t*) cannot be continuously differentiable, hence we assumed *s* to be a function of bounded variation. We proved that $\frac{ds}{dt}$ and *c* must be continuous with respect to Lebesgue measure in (0, *T*) but that there may be a gulp of consumption at either or both endpoints. Since such gulps take place in continuous time, they are not artifacts of discrete time and could be observed experimentally.

We described how to construct optimal solutions.

In Section 6.1, we considered solutions under the assumptions of a high discount rate (assumptions (33) and (41)). This happens in societies characterized by high conflict. The solution we obtained for that case can be described as follows: It is optimal to consume all of one’s wealth at once and not wait, no matter what one’s utility function is. Indeed, in societies with low safety levels, when life expectancy is short (e.g., during famines in ancient times), or when an agent expects to lose his wealth to robbers, it seems reasonable to consume all of the wealth at once. This conclusion is independent of the time horizon.

The analysis of consumption with satiation when the discount rate is low (conditions (32) and (40)) is more complicated. It turns out that a gulp of consumption at the end of the consumption period is always optimal (see Proposition 14), because no further penalty can be imposed by the growth of satiation. In fact, there exists anecdotal evidence of addicts having final binges before going into rehab (as in the movie “The Wonderful Whites of Virginia”), smokers taking the last puff before quitting (as in the movie “A Good Woman”), and death-row inmates accepting the offer of the last wish. According to [19], empirical data indicate that such gulps of consumption take place when people consume cultural goods, such as music. Detailed analysis in Section 7.4 shows that when the wealth to be consumed is small and/or satiation is high, the agent consumes all of his wealth at the very end of the consumption period. When the wealth is large and/or satiation is low, the agent consumes some portion in a gulp of consumption at the beginning of his consumption period, as shown in Section 6.2. This can be explained as follows: Wealthy agents consume a lot at the beginning in order to curtail their desires by increasing their satiation level, and then they consume at a moderate rate throughout the rest of their consumption period. They also leave some wealth to be consumed at the very end. There is also an intermediate pattern, where an agent begins consumption after some delay and then enjoys a gulp of consumption at the very end of his consumption interval. Although the terminal gulp of consumption is non-negotiable in our model, we rarely observe it in the real world. This is especially true when non-cultural goods are consumed. A possible explanation is that agents are not aware of the exact time at which their consumption life will end, and as a result they optimize beyond the actual time of termination of their consumption. The finding regarding the optimality of the terminal gulp of consumption may explain the large sums some people spend on funerals and funerary monuments. One extreme example of such behavior is that of Caterina Campodonico, who lived in Italy in the 19th century and is said to have saved for most of her life in order to afford an elaborate funerary monument in the famous Staglieno Cemetery in Genoa; see [20]. Leaving an inheritance to descendants could also be interpreted as a gulp of consumption at the end of the consumption period.
